# Respiratory Distress in the Pediatric ED: A Case-based Self-directed Learning Module

**DOI:** 10.21980/J8T64M

**Published:** 2022-10-15

**Authors:** Sravana Paladugu, Ngoc Van Horn, Christine Kulstad

**Affiliations:** * University of Texas Southwestern Medical Center, Department of Emergency Medicine, Dallas, TX; ^ University of Texas Southwestern Medical Center, Department of Pediatrics, Dallas, TX

## Abstract

**Audience:**

Emergency medicine residents, pediatric residents on an EM rotation

**Introduction:**

Emergency medicine residents are expected to recognize and treat patients of all ages and diseases of all varieties, yet most education and training is focused on the adult patient. Exposure to pediatrics is generally integrated into training across all years of residency, but time spent in the pediatric emergency department is still a small portion of resident education. This module aims to enhance the ability of the emergency medicine residents to recognize and treat respiratory distress in children, one of the most common presenting chief complaints in the pediatric population, by integrating the concepts of case-based learning, self-directed learning and self-testing.

**Educational Objectives:**

By the end of this module, learners will be able to: 1) recognize the unique pathophysiology for respiratory distress in the pediatric population and formulate a broad differential; 2) understand the treatment principles for the most common causes of respiratory distress in children; 3) navigate and apply validated clinical decision-making tools for treatment of pediatric respiratory illnesses.

**Educational Methods:**

A learning module consisting of six clinical vignettes based on the most common causes of respiratory distress in children, with associated self-test questions, and key learning concepts was created for resident education. This module was a self-directed PowerPoint slideshow with embedded questions and links to evidence-based clinical decision-making tools.

**Research Methods:**

A survey was created to gauge the residents’ perceptions of the learning module and its usefulness in their learning.

**Results:**

Twenty (30%) residents used this module and took the survey. Ninety percent of respondents felt more comfortable managing respiratory distress in children after completing this module. Ninety-five percent of respondents felt they had sufficient knowledge of the topic after completing the module and would like to have more modules such as this one on other topics.

**Discussion:**

Residents indicated in the survey that the module enhanced their knowledge and comfort with clinical practice. This unique learning module integrates basic and clinical sciences and utilizes many different learning concepts to engage and motivate the adult learner. The module may also be re-created in order to cover other similar topics as a supplement to resident education.

**Topics:**

Pediatrics, respiratory, infectious disease, asthma, croup, anaphylaxis, foreign body aspiration, bronchiolitis, laryngomalacia.

## USER GUIDE

**Table:** 

**List of Resources:**	
Abstract	1
User Guide	3
Appendix A: Respiratory Distress in the Pediatric ED: Case-based Self-directed Learning Module PowerPoint	6

**Learner Audience:**

Interns, Junior Residents, Pediatric Residents on EM rotations

**Time Required for Implementation:**

Learners are expected to complete the module in about 45 minutes, asynchronously.

**Recommended Number of Learners per Instructor:** This is a stand-alone learning module that is designed to be completed asynchronously by residents. Instructor to learner ratios do not apply.

**Topics:**

Pediatrics, respiratory, infectious disease, asthma, croup, anaphylaxis, foreign body aspiration, bronchiolitis, laryngomalacia.

**Objectives:**

By the end of this module, learners will be able to:

1. Recognize the presentation of laryngomalacia, and need for urgent versus outpatient consultation.
2. List appropriate treatment strategies for bronchiolitis.
3. Recognize the presentation of foreign body aspiration and outline a strategy for diagnosis.
4. Describe the emergent treatment of anaphylaxis.
5. Distinguish between mild and more serious presentations of croup, and choose corresponding treatments.

### Linked objectives and methods

To solidify knowledge of respiratory distress in children, a cased-based learning (CBL) module was designed for use by EM residents as an adjunct to clinical training, formal lectures, and textbooks. CBL is a pedagogical method that is becoming more and more prevalent in medical education^1^. It involves the use of clinical vignettes built on real or imagined patient cases to teach and solidify basic science principles. CBL has been described as a way to connect the basic sciences to clinical science, thus achieving the concept of vertical integration^2^. Vertical integration is beneficial for the adult learner in that it provides a context for learning and is also motivational in nature, the ultimate motivation being the ability to contribute to patient care.^3^ In motivating learners to be active participant in their learning, CBL and vertical integration incorporate the idea of inquiry-based learning, in which it is the responsibility of the student to identify knowledge gaps and then carry out a self-directed learning process of seeking out the required information^4^. Not only is CBL shown to promote a deeper level of learning and higher order thinking, but it is also enjoyed by both learners and teachers due to its ability to engage the student.^1^,^2^

There is more information available at our fingertips now than ever before, and it can be difficult for a learner to sort through this vast amount of data and resources in order to find something accurate and concise. For this reason, we created a learning module that incorporates several evidence-based and validated resources that provide the necessary clinical decision-making guidelines. Healthcare professionals should be aware of the existence of these guidelines and know to how to find and navigate these clinical decision-making tools because this is an important aspect of practicing evidence-based medicine.

This module was emailed to all residents as an optional activity, for which they would qualify for asynchronous learning credit. After the email, a link to the module was placed on the residency’s MedHub home page for easy access. There was no requirement to complete it, or time limit in which to do so.

### Recommended pre-reading for instructor

Because this is a self-directed module, no instructor is required.

### Results and Tips for Successful Implementation

Out of a group of 66 total emergency medicine residents, 20 (30%) residents accessed and completed the learning module and survey. Of the 20 residents that took the survey, 12 (60%) were first year residents, 6 (30%) were second year residents, and 2 (10%) were third year residents. The majority of feedback from the survey was positive. Eighty percent of residents strongly agreed, and 20% of residents agreed, that the learning module was easy to navigate. Before completing the learning module, 60% of residents either strongly agreed or agreed that they had sufficient knowledge of the topic, which increased to 95% of residents after completing the learning module. Similarly 30% of residents strongly agreed or agreed that they felt comfortable managing respiratory distress in children before completing the learning module, which increased to 90%. Overall, 95% of residents either strongly agreed or agreed that they would like to have more learning modules such as this one on other similar topics. See Table 1 for answers to each question.

This module was not mandatory, and we saw a relatively low use of it. To improve implementation, requiring completion of the module for asynchronous learning credit would likely improve its utility.

### Associated content (optional)

Appendix A: Respiratory Distress in the Pediatric ED: Case-based Self-directed Learning Module PowerPoint

### Learner responsible content (LRC, optional)

N/A. Links to evidence-based clinical decision aids included in the module.

### Technology necessary

Computer or tablet capable of viewing a PowerPoint slideshow, with internet connection to follow links as desired by the learner.

### Assessment (optional)

Contained in the self-directed learning module are a series of self-assessment questions.

## Figures and Tables

**Table 1:** Survey Results (N=20)

%					
Question	Strongly disagree	Disagree	Neutral	Agree	Strongly agree
Q1. The module was easy to navigate through	0	0	0	20	80
Q2. I had sufficient knowledge of the topic before completing the learning module	5	5	30	50	10
Q3. I have sufficient knowledge of the topic after completing the learning module	0	0	5	40	55
Q4. I felt comfortable managing respiratory distress in children before completing this module	5	25	40	20	10
Q5. I feel comfortable managing respiratory distress in children after completing this module	0	0	10	65	25
Q6. I would like to have more learning modules such as this one on other similar topics	0	0	5	40	55

## USER GUIDE AND LEARNER MATERIALS

### Appendix A Respiratory Distress in the Pediatric ED: Case-based Self-directed Learning Module PowerPoint

Please see associated PowerPoint file
