# Supplementary material for: Respiratory Distress in the Pediatric ED: A Case-based Self-directed Learning Module
Source: J Educ Teach Emerg Med. 2022 Oct 15;7(4):L1–6. doi: 10.21980/J8T64M (PMC10332669; doi:10.21980/J8T64M)
Supplement: Supplementary file 1 — Please see associated PowerPoint file [file JETem-7-4-L1-supp1.pptx]

## Slide 1
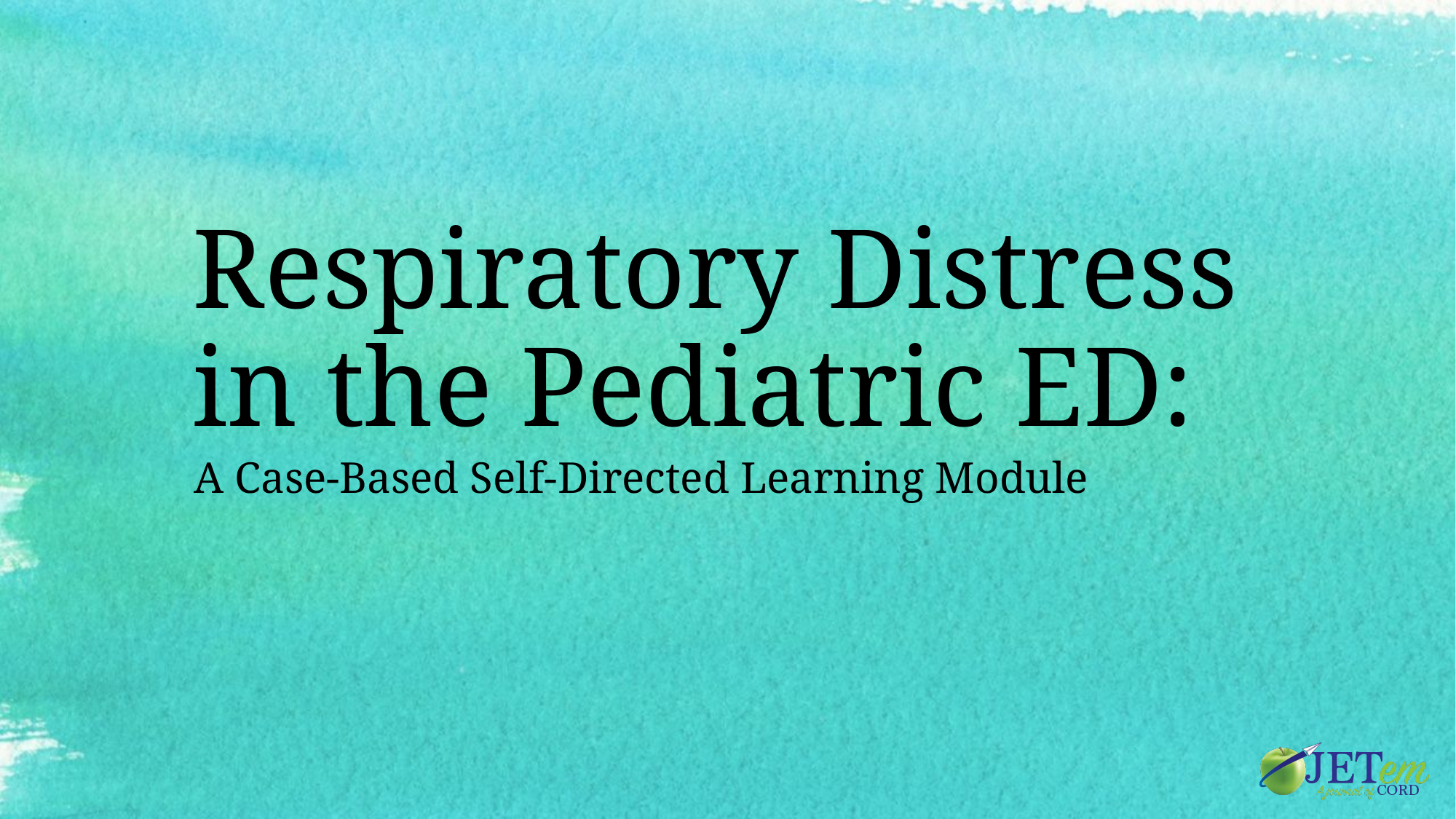

# Respiratory Distress in the Pediatric ED:
A Case-Based Self-Directed Learning Module

## Slide 2
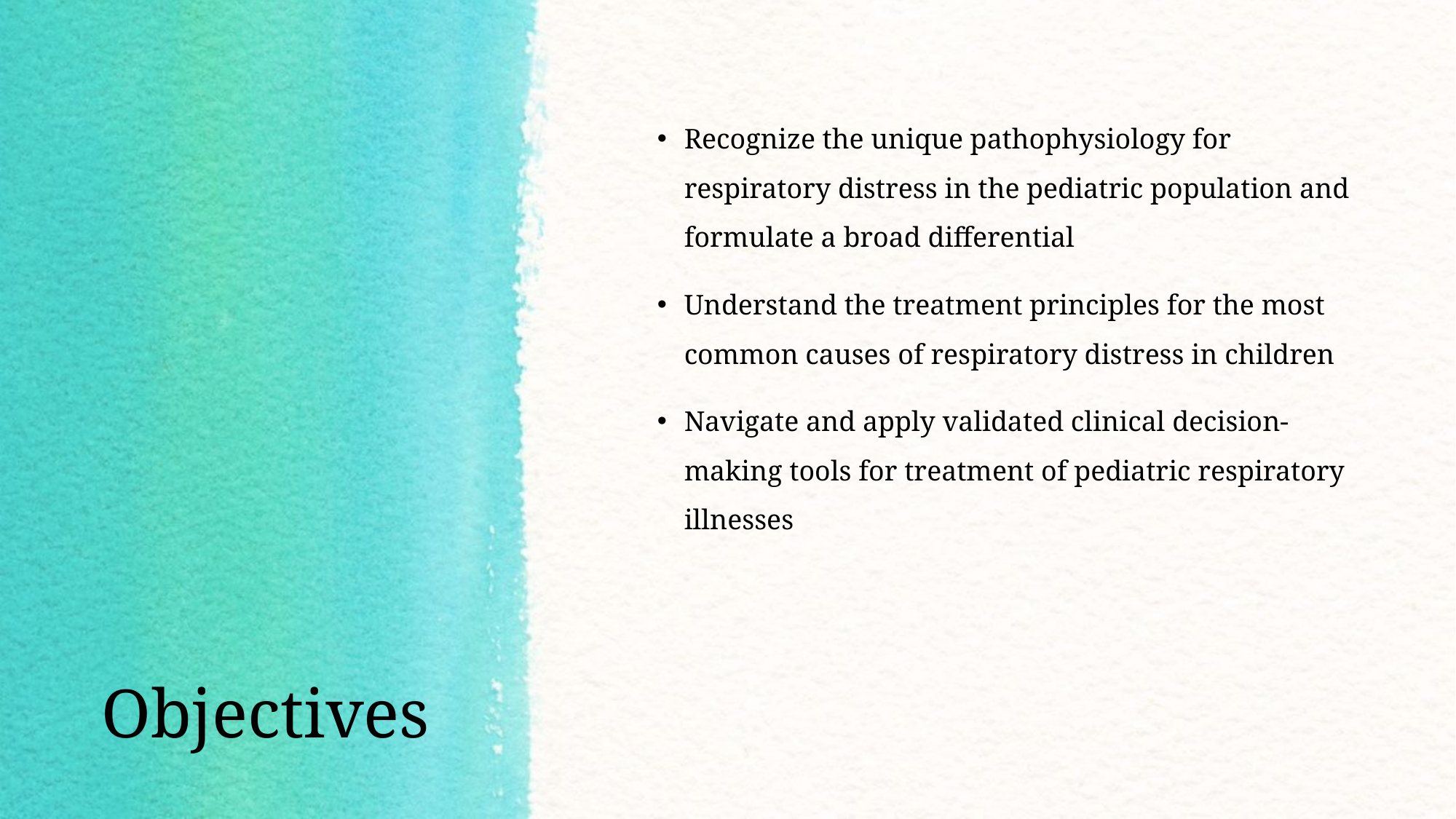

Recognize the unique pathophysiology for respiratory distress in the pediatric population and formulate a broad differential
Understand the treatment principles for the most common causes of respiratory distress in children
Navigate and apply validated clinical decision-making tools for treatment of pediatric respiratory illnesses
# Objectives

## Slide 3
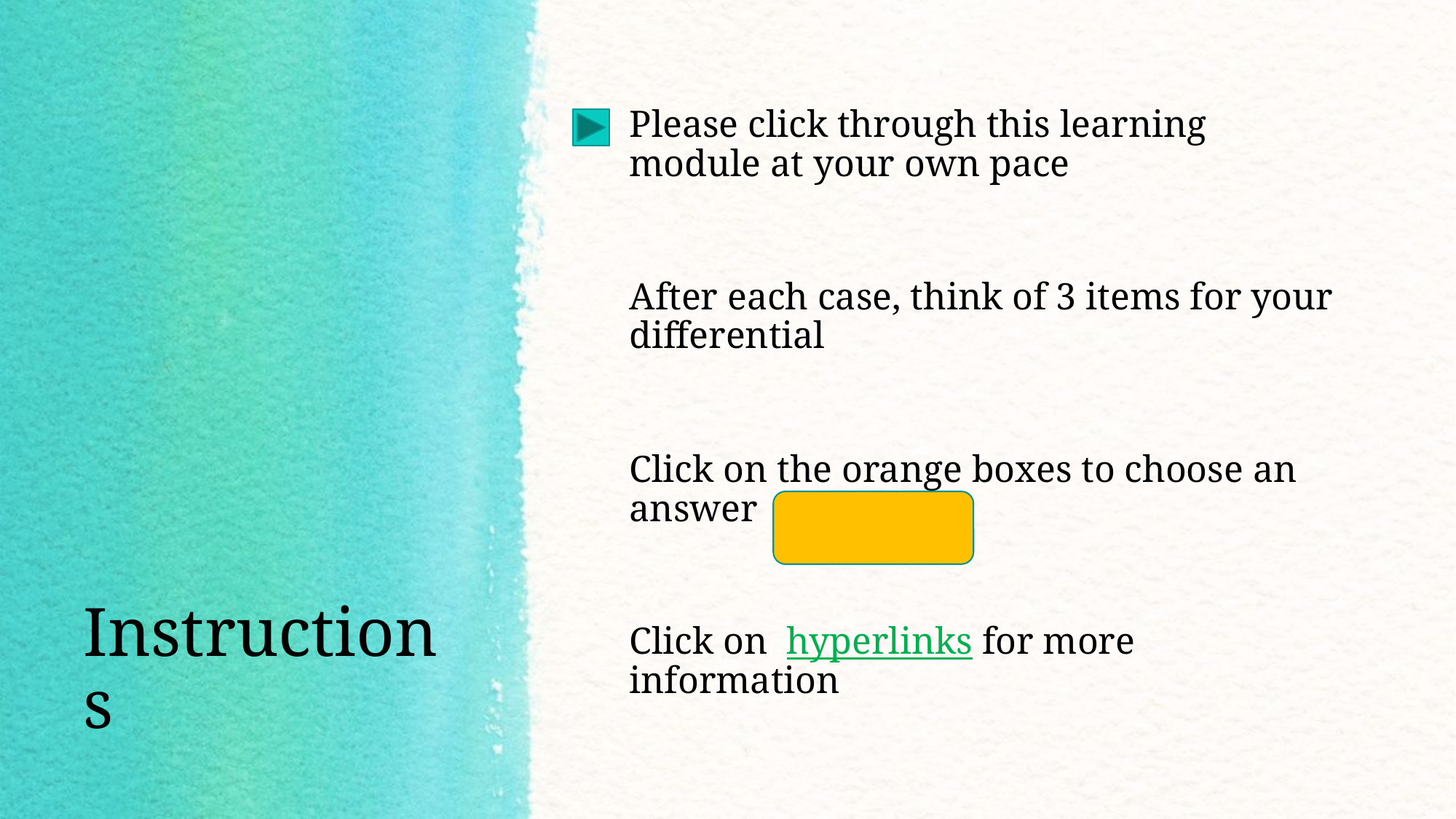

Please click through this learning module at your own pace
After each case, think of 3 items for your differential
Click on the orange boxes to choose an answer
Click on hyperlinks for more information
# Instructions

## Slide 4
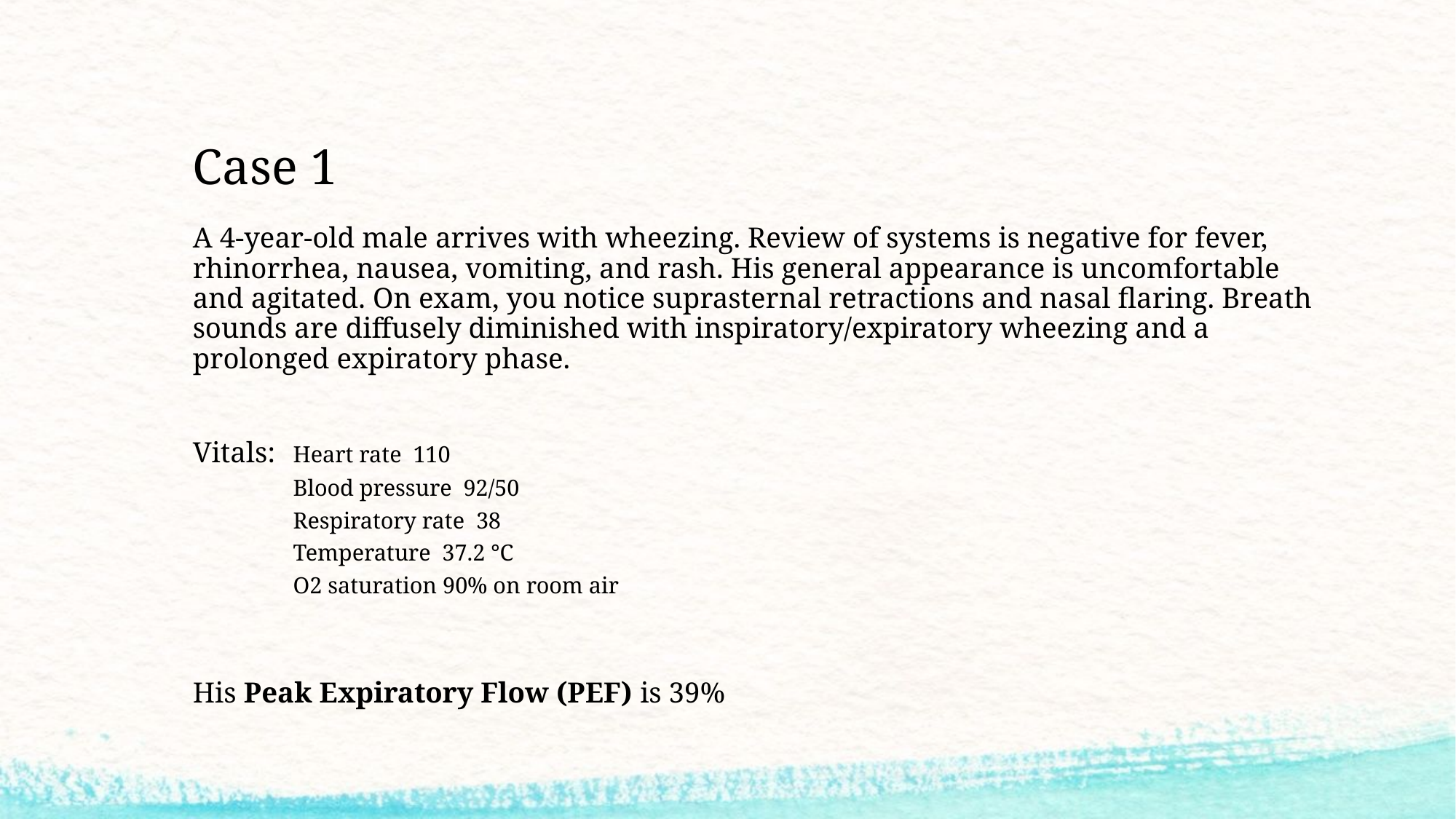

# Case 1
A 4-year-old male arrives with wheezing. Review of systems is negative for fever, rhinorrhea, nausea, vomiting, and rash. His general appearance is uncomfortable and agitated. On exam, you notice suprasternal retractions and nasal flaring. Breath sounds are diffusely diminished with inspiratory/expiratory wheezing and a prolonged expiratory phase.
Vitals: 	Heart rate 110
	Blood pressure 92/50
	Respiratory rate 38
	Temperature 37.2 °C
	O2 saturation 90% on room air
His Peak Expiratory Flow (PEF) is 39%

## Slide 5
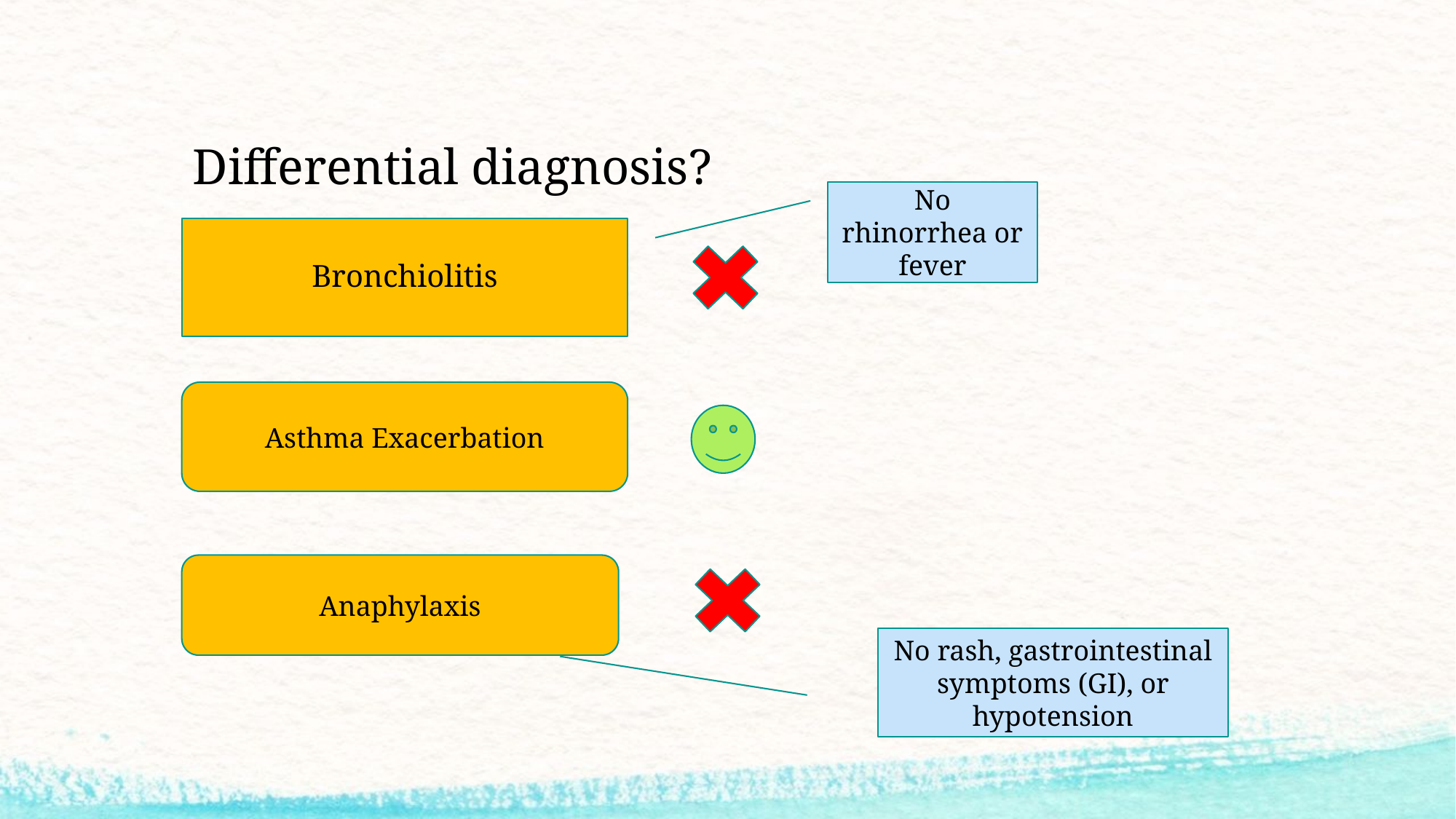

# Differential diagnosis?
No rhinorrhea or fever
Bronchiolitis
Asthma Exacerbation
Anaphylaxis
No rash, gastrointestinal symptoms (GI), or hypotension

## Slide 6
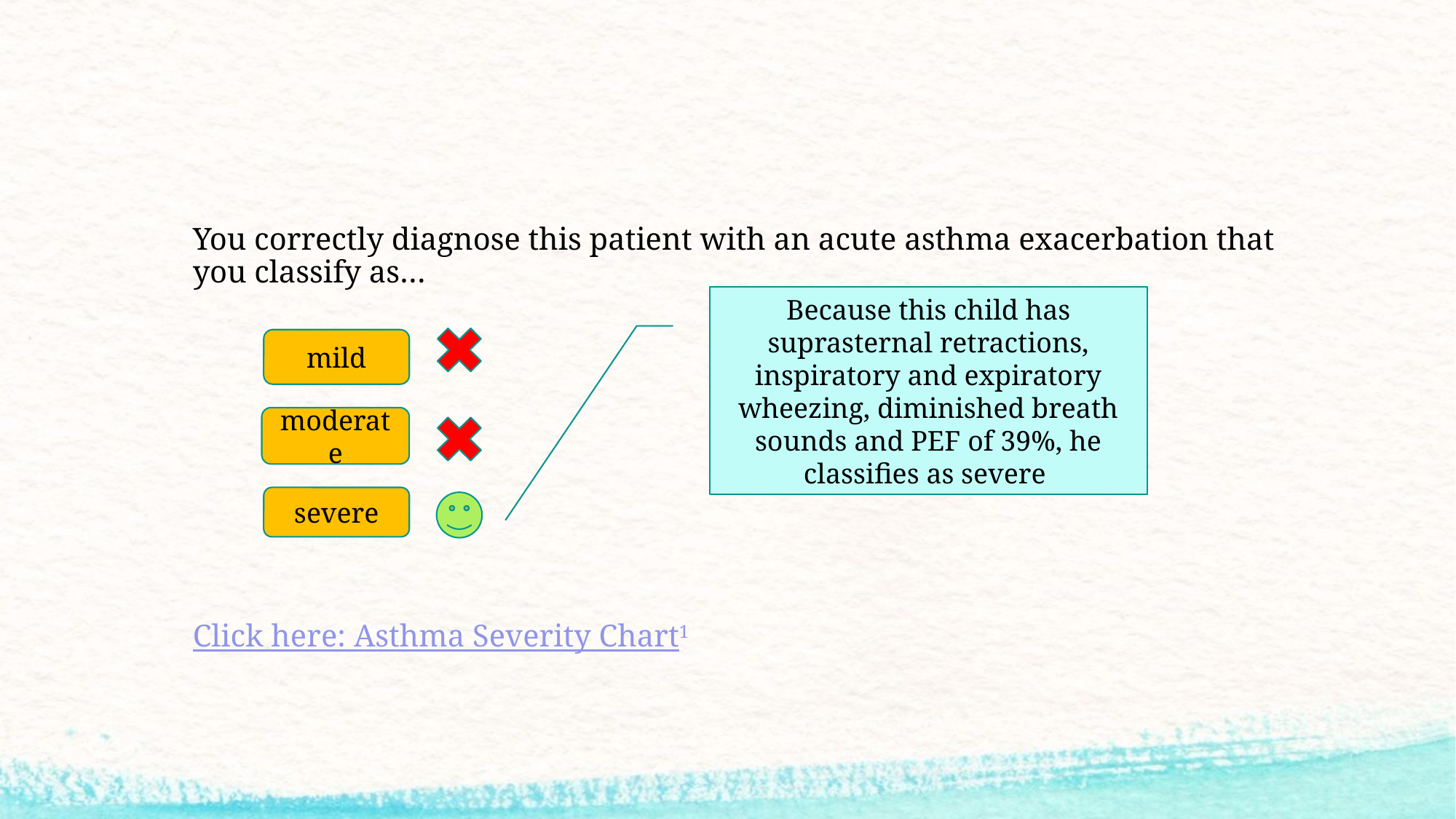

You correctly diagnose this patient with an acute asthma exacerbation that you classify as…
Click here: Asthma Severity Chart1
Because this child has suprasternal retractions, inspiratory and expiratory wheezing, diminished breath sounds and PEF of 39%, he classifies as severe
mild
moderate
severe

## Slide 7
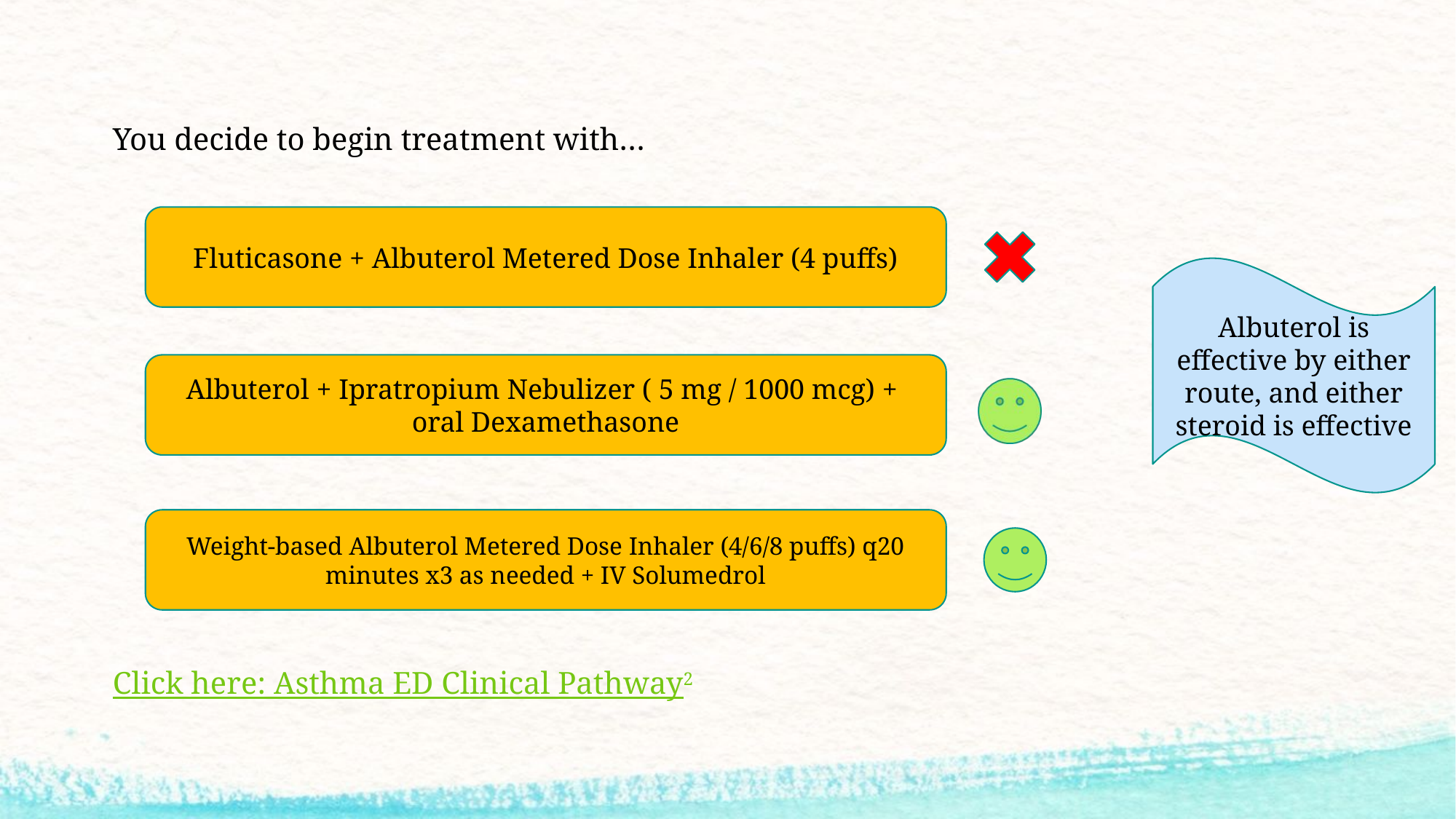

You decide to begin treatment with…
Click here: Asthma ED Clinical Pathway2
Fluticasone + Albuterol Metered Dose Inhaler (4 puffs)
Albuterol is effective by either route, and either steroid is effective
Albuterol + Ipratropium Nebulizer ( 5 mg / 1000 mcg) +
oral Dexamethasone
Weight-based Albuterol Metered Dose Inhaler (4/6/8 puffs) q20 minutes x3 as needed + IV Solumedrol

## Slide 8
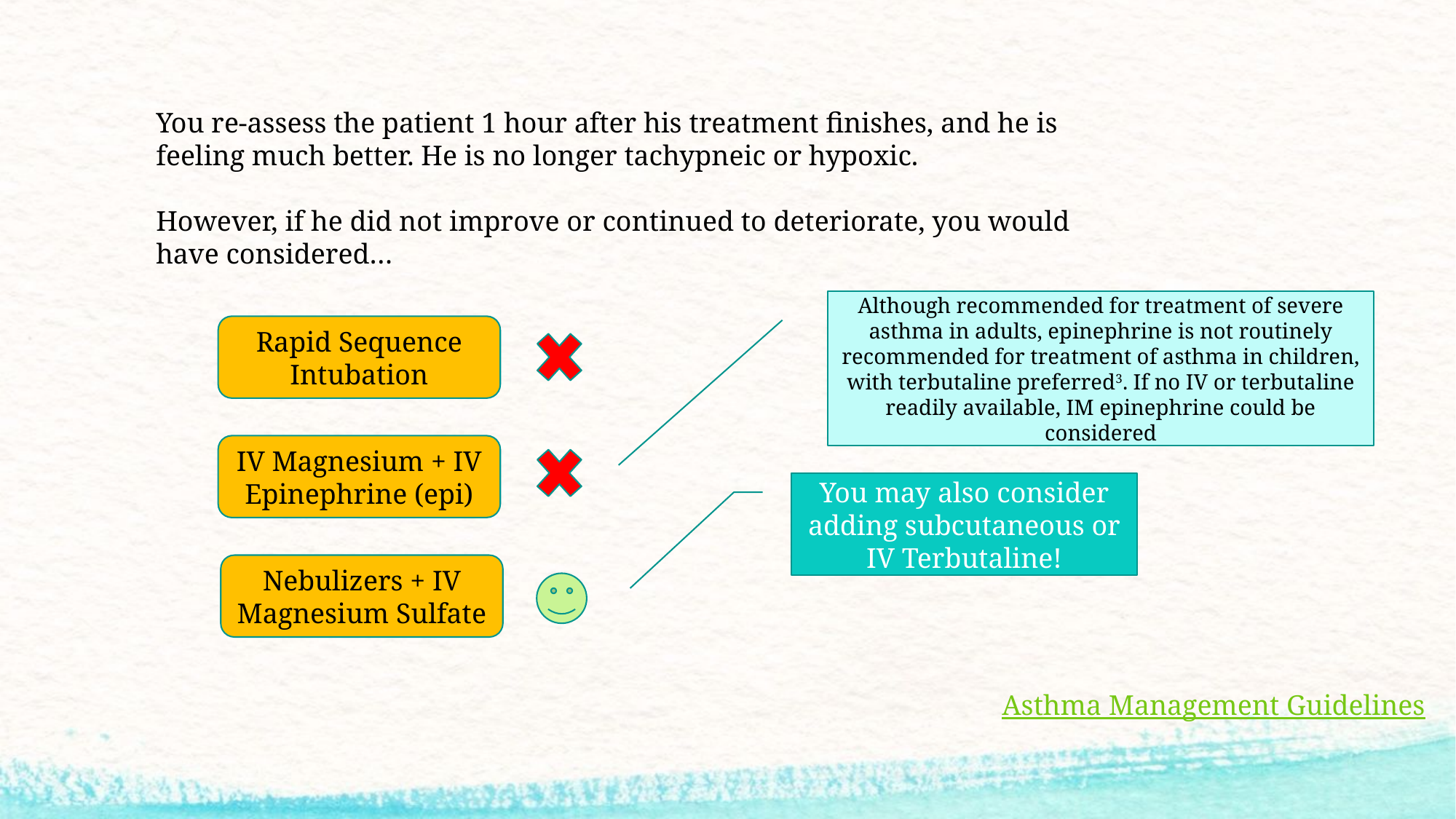

You re-assess the patient 1 hour after his treatment finishes, and he is feeling much better. He is no longer tachypneic or hypoxic.
However, if he did not improve or continued to deteriorate, you would have considered…
Although recommended for treatment of severe asthma in adults, epinephrine is not routinely recommended for treatment of asthma in children, with terbutaline preferred3. If no IV or terbutaline readily available, IM epinephrine could be considered
Rapid Sequence Intubation
IV Magnesium + IV Epinephrine (epi)
You may also consider adding subcutaneous or IV Terbutaline!
Nebulizers + IV Magnesium Sulfate
Asthma Management Guidelines

## Slide 9
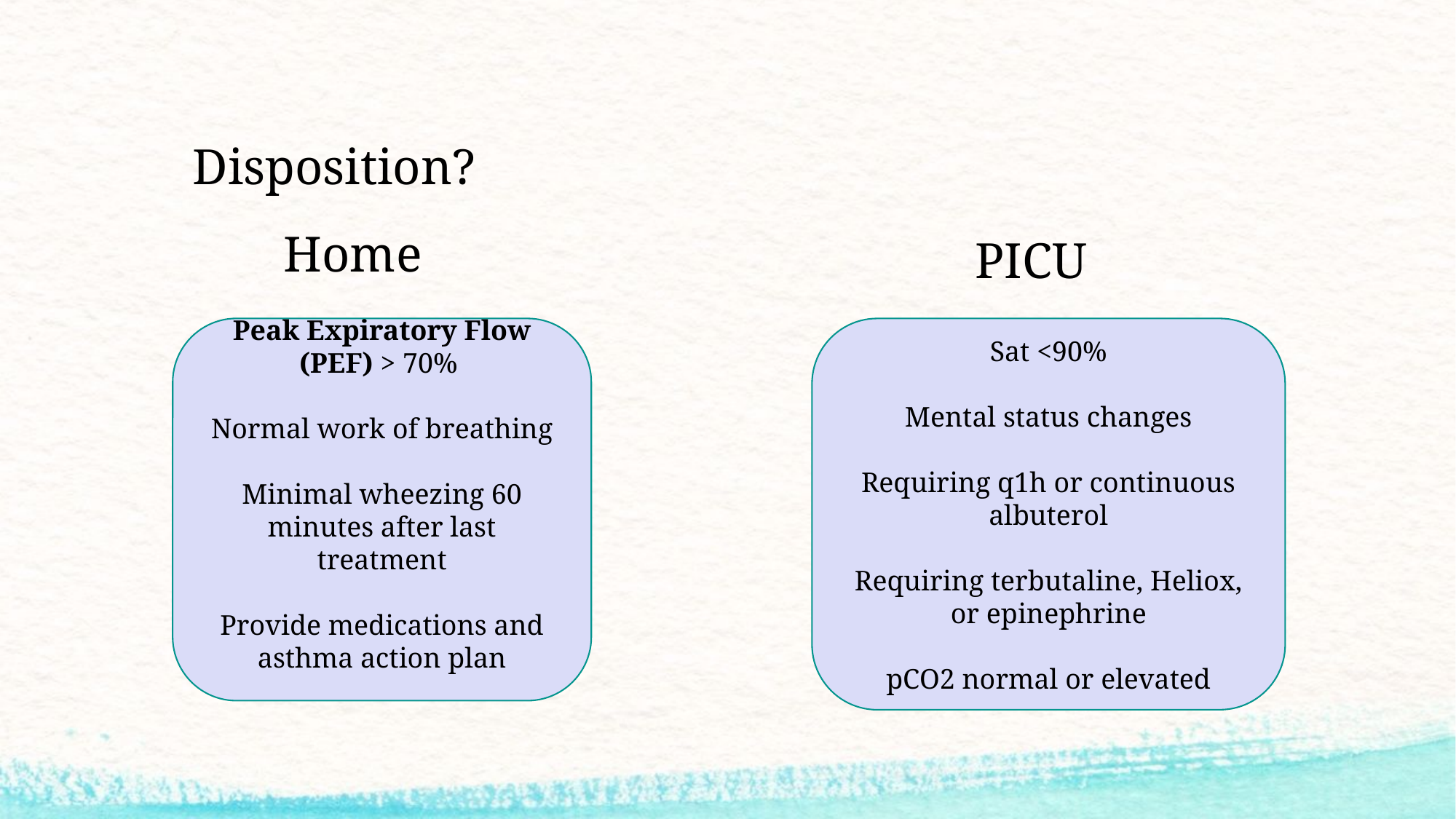

# Disposition?
Home
PICU
Peak Expiratory Flow (PEF) > 70%
Normal work of breathing
Minimal wheezing 60 minutes after last treatment
Provide medications and asthma action plan
Sat <90%
Mental status changes
Requiring q1h or continuous albuterol
Requiring terbutaline, Heliox, or epinephrine
pCO2 normal or elevated

## Slide 10
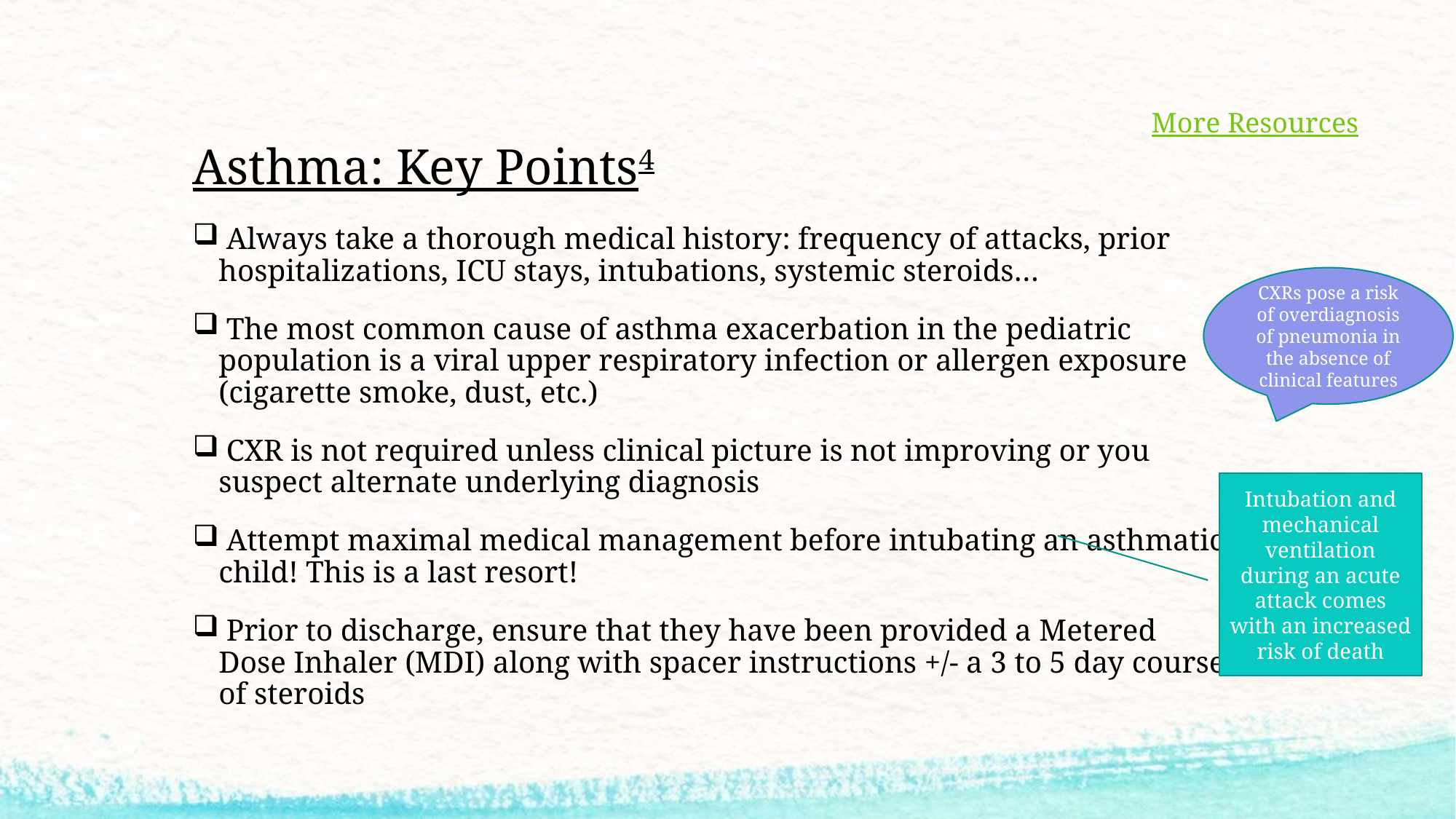

# Asthma: Key Points4
More Resources
 Always take a thorough medical history: frequency of attacks, prior hospitalizations, ICU stays, intubations, systemic steroids…
 The most common cause of asthma exacerbation in the pediatric population is a viral upper respiratory infection or allergen exposure (cigarette smoke, dust, etc.)
 CXR is not required unless clinical picture is not improving or you suspect alternate underlying diagnosis
 Attempt maximal medical management before intubating an asthmatic child! This is a last resort!
 Prior to discharge, ensure that they have been provided a Metered Dose Inhaler (MDI) along with spacer instructions +/- a 3 to 5 day course of steroids
CXRs pose a risk of overdiagnosis of pneumonia in the absence of clinical features
Intubation and mechanical ventilation during an acute attack comes with an increased risk of death

## Slide 11
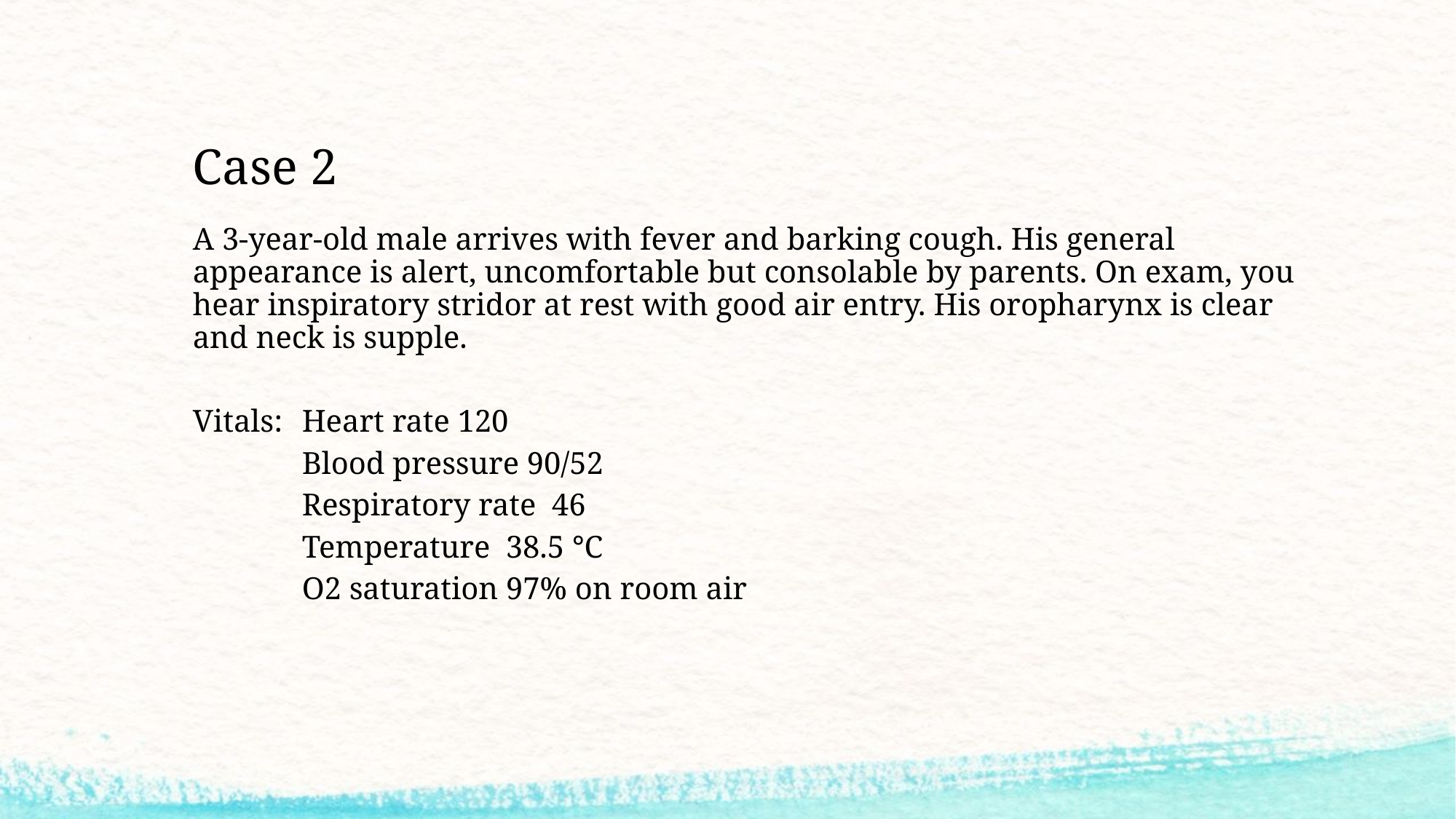

# Case 2
A 3-year-old male arrives with fever and barking cough. His general appearance is alert, uncomfortable but consolable by parents. On exam, you hear inspiratory stridor at rest with good air entry. His oropharynx is clear and neck is supple.
Vitals: 	Heart rate 120
	Blood pressure 90/52
	Respiratory rate 46
	Temperature 38.5 °C
	O2 saturation 97% on room air

## Slide 12
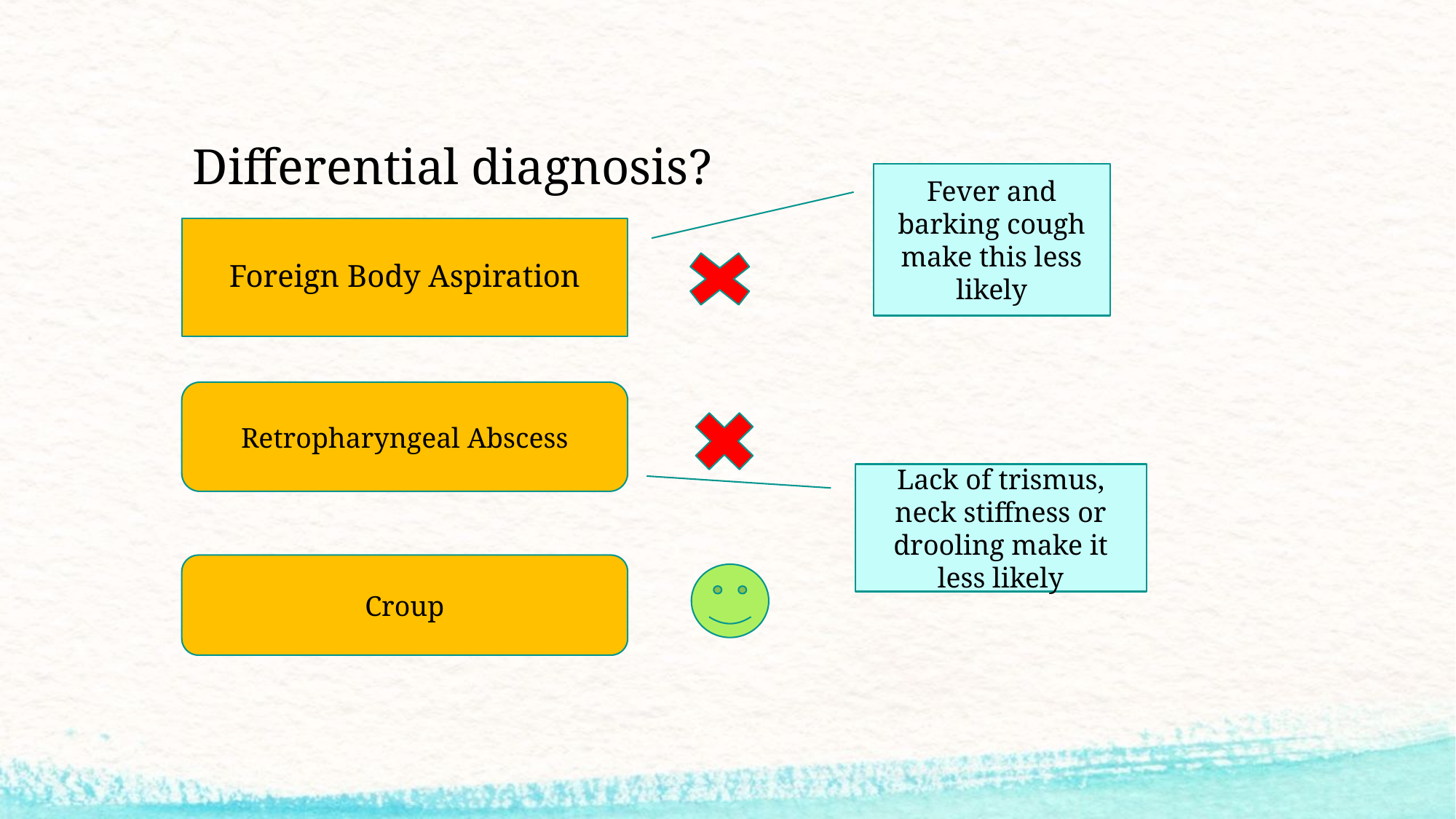

# Differential diagnosis?
Fever and barking cough make this less likely
Foreign Body Aspiration
Retropharyngeal Abscess
Lack of trismus, neck stiffness or drooling make it less likely
Croup

## Slide 13
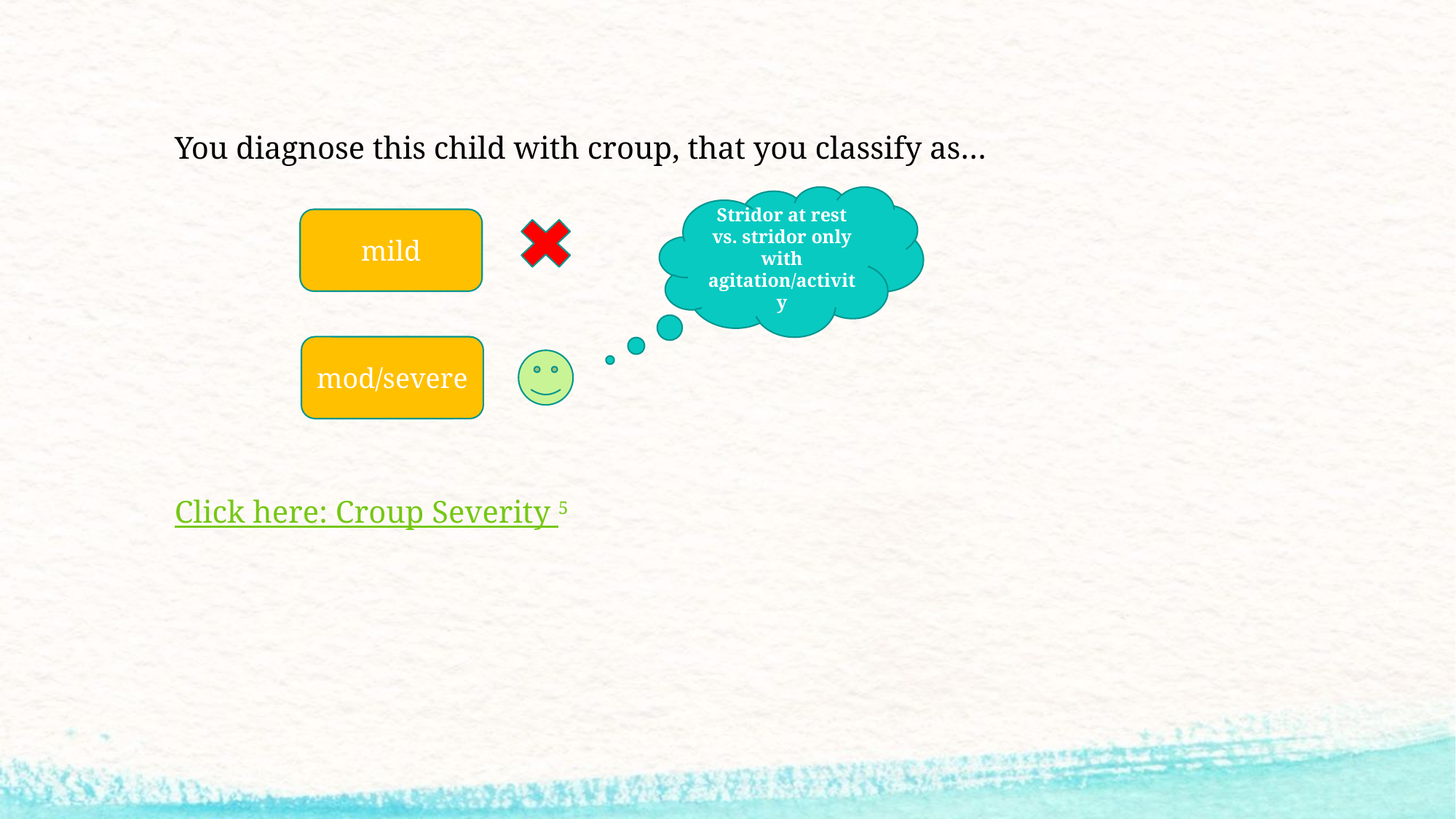

You diagnose this child with croup, that you classify as…
Click here: Croup Severity 5
Stridor at rest vs. stridor only with agitation/activity
mild
mod/severe

## Slide 14
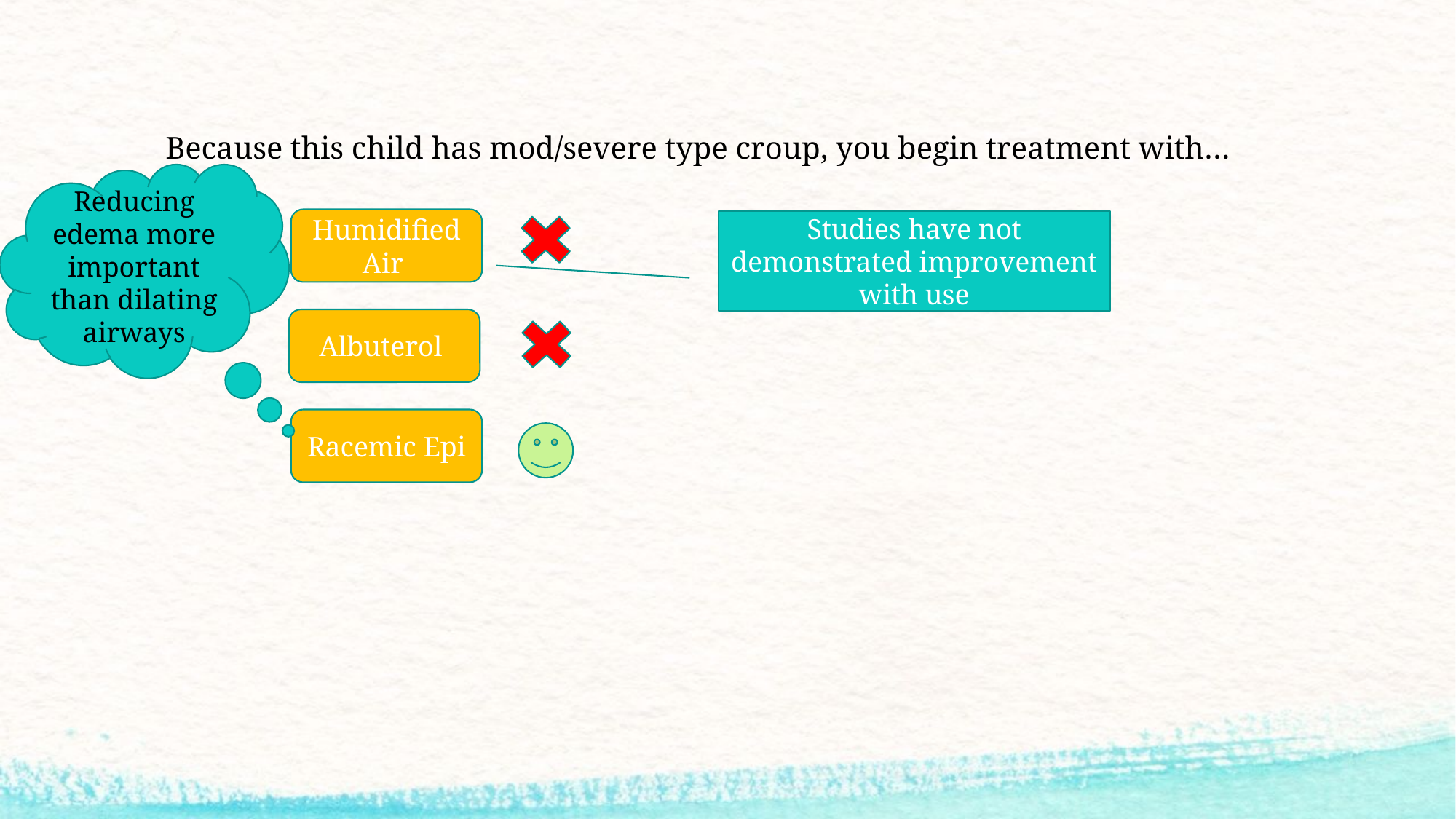

Because this child has mod/severe type croup, you begin treatment with…
Reducing edema more important than dilating airways
Humidified Air
Studies have not demonstrated improvement with use
Albuterol
Racemic Epi

## Slide 15
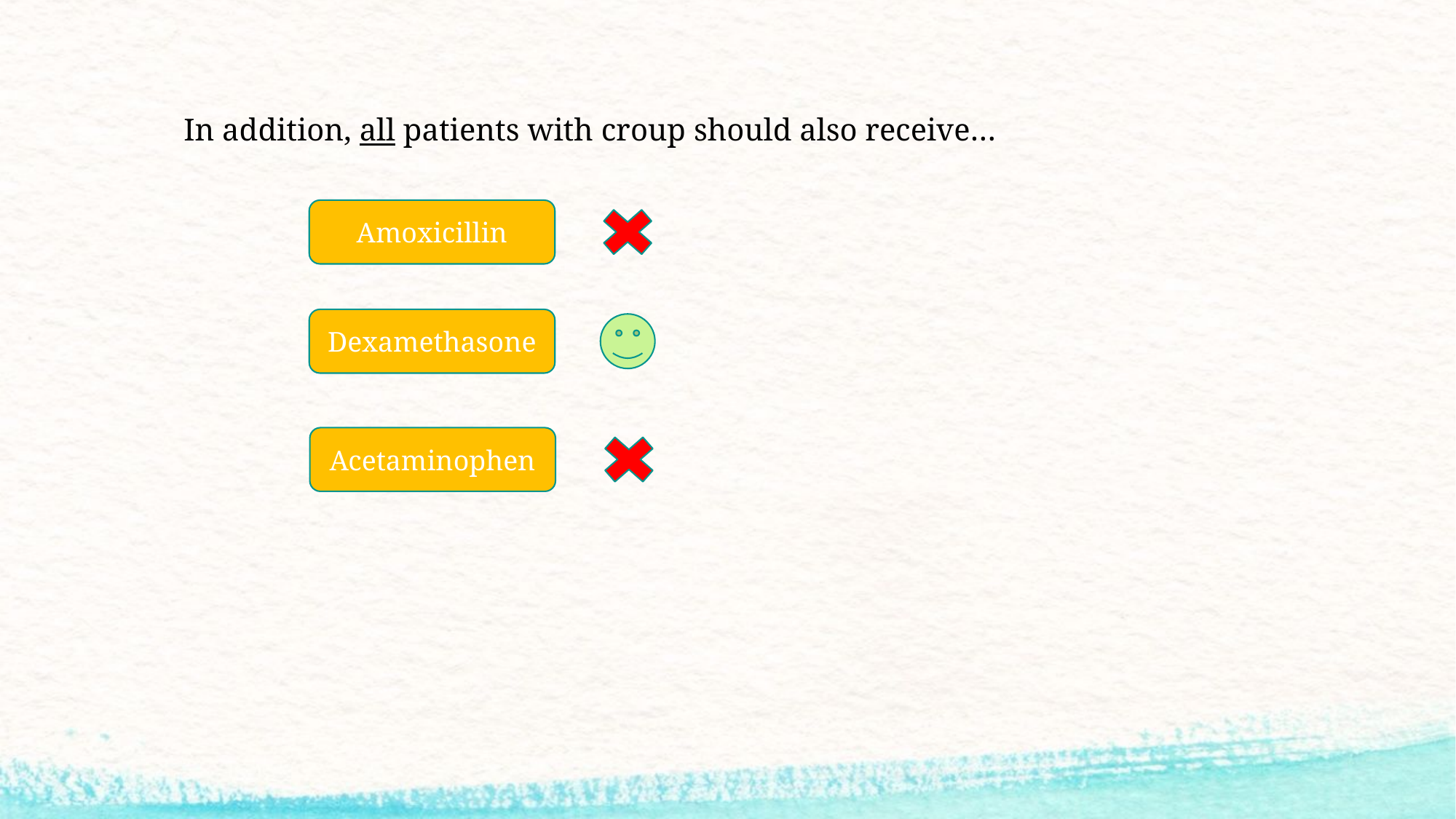

In addition, all patients with croup should also receive…
Amoxicillin
Dexamethasone
Acetaminophen

## Slide 16
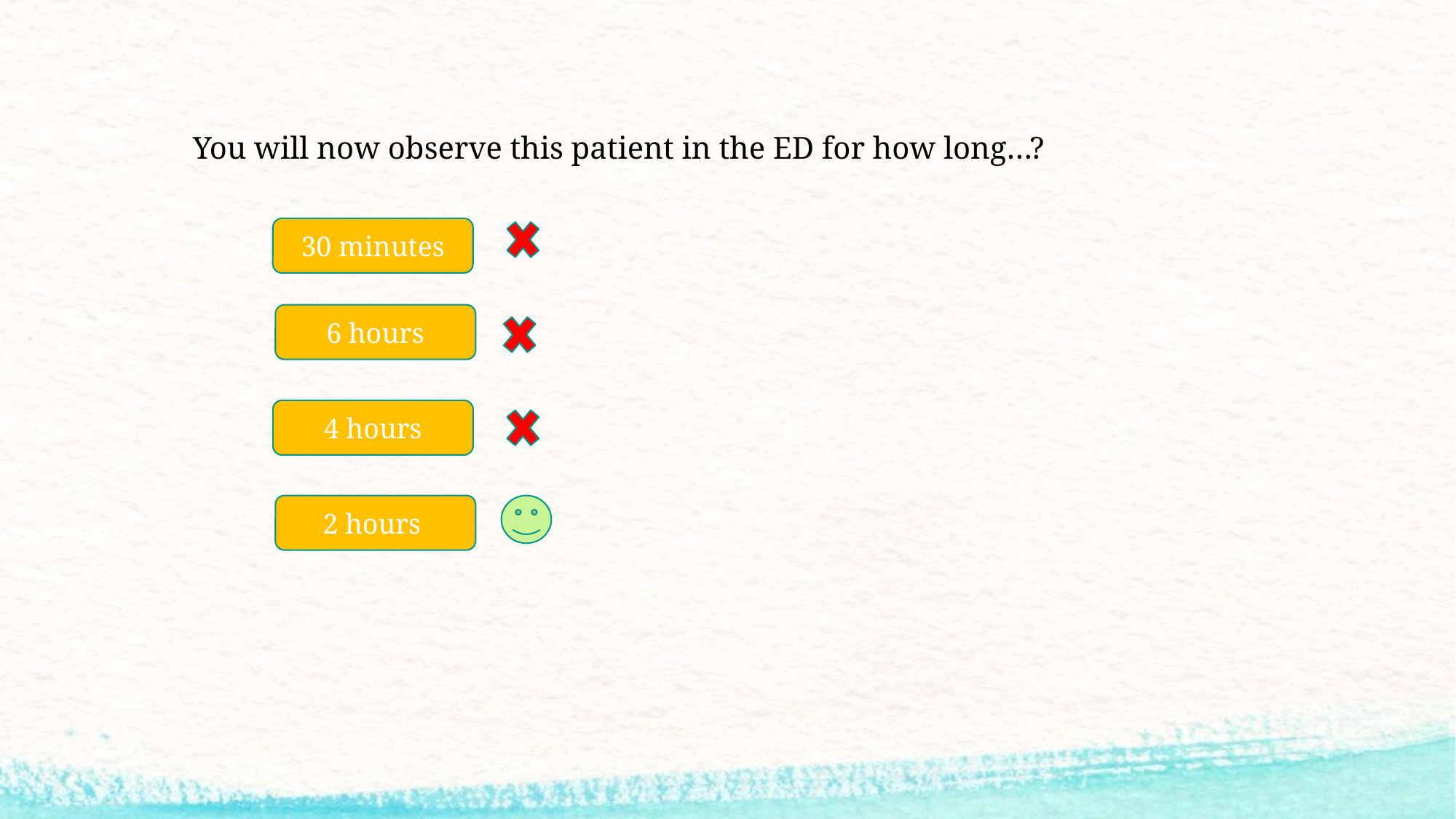

You will now observe this patient in the ED for how long…?
30 minutes
6 hours
4 hours
2 hours

## Slide 17
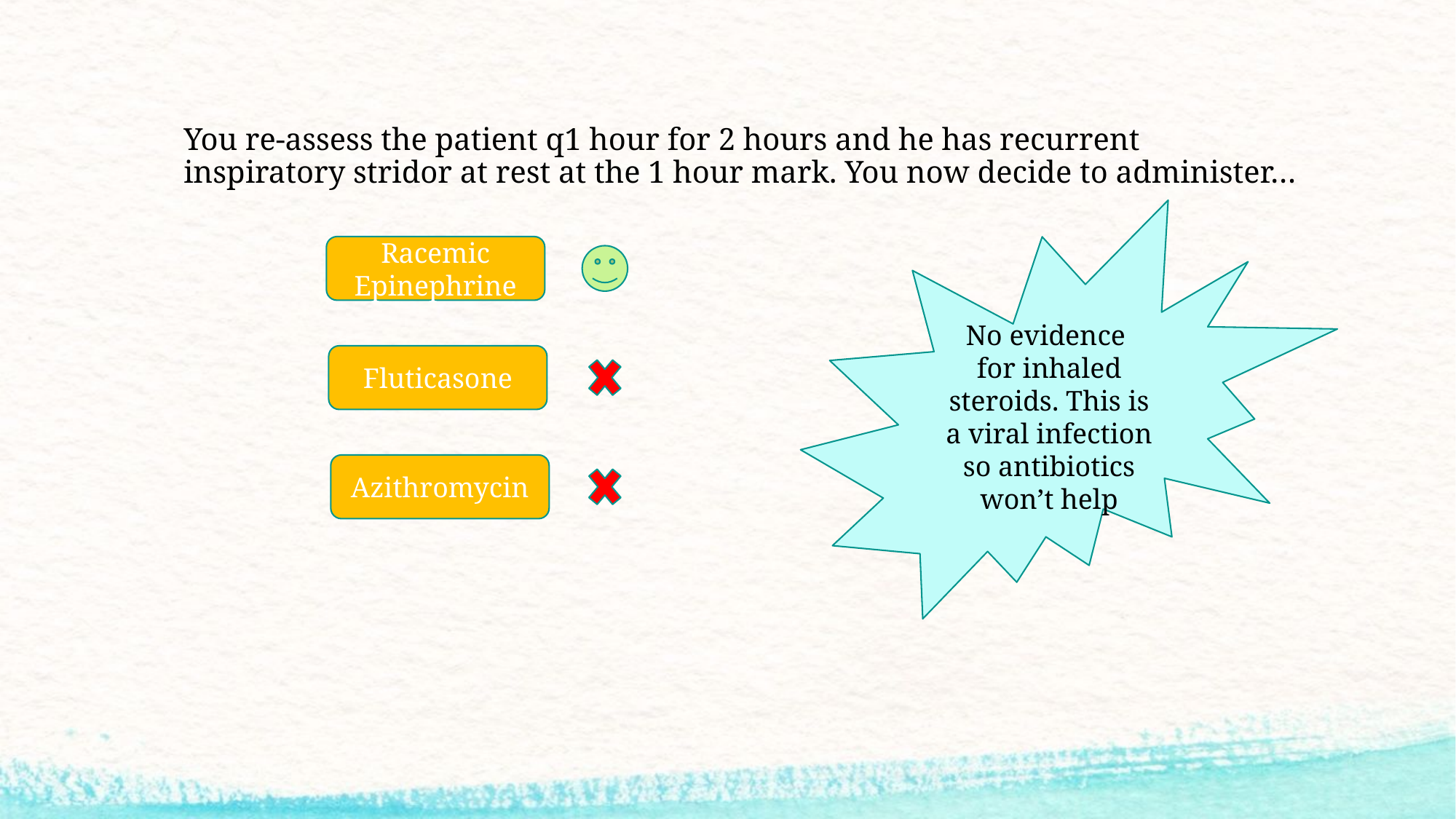

You re-assess the patient q1 hour for 2 hours and he has recurrent inspiratory stridor at rest at the 1 hour mark. You now decide to administer…
No evidence for inhaled steroids. This is a viral infection so antibiotics won’t help
Racemic Epinephrine
Fluticasone
Azithromycin

## Slide 18
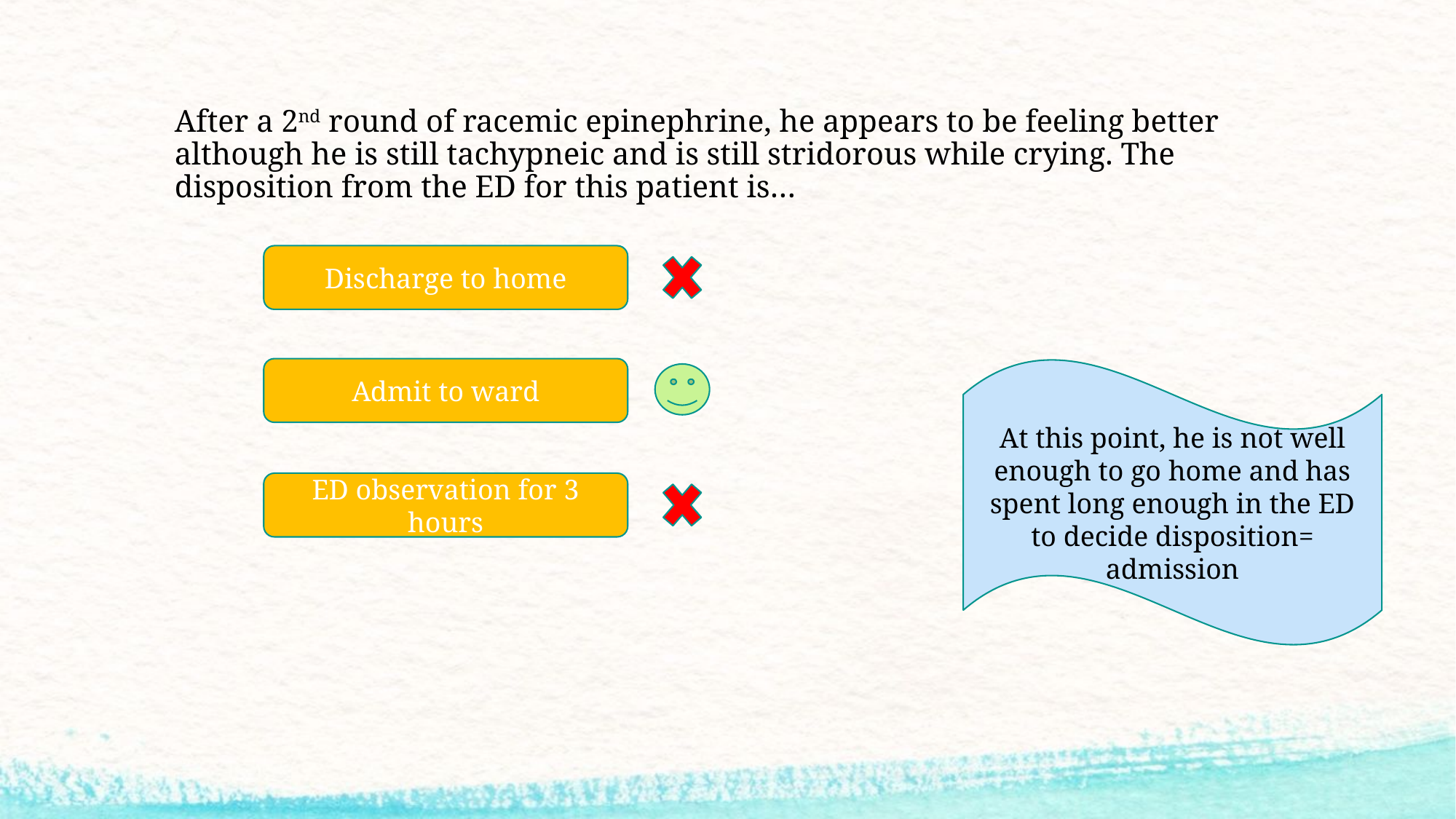

After a 2nd round of racemic epinephrine, he appears to be feeling better although he is still tachypneic and is still stridorous while crying. The disposition from the ED for this patient is…
Discharge to home
Admit to ward
At this point, he is not well enough to go home and has spent long enough in the ED to decide disposition= admission
ED observation for 3 hours

## Slide 19
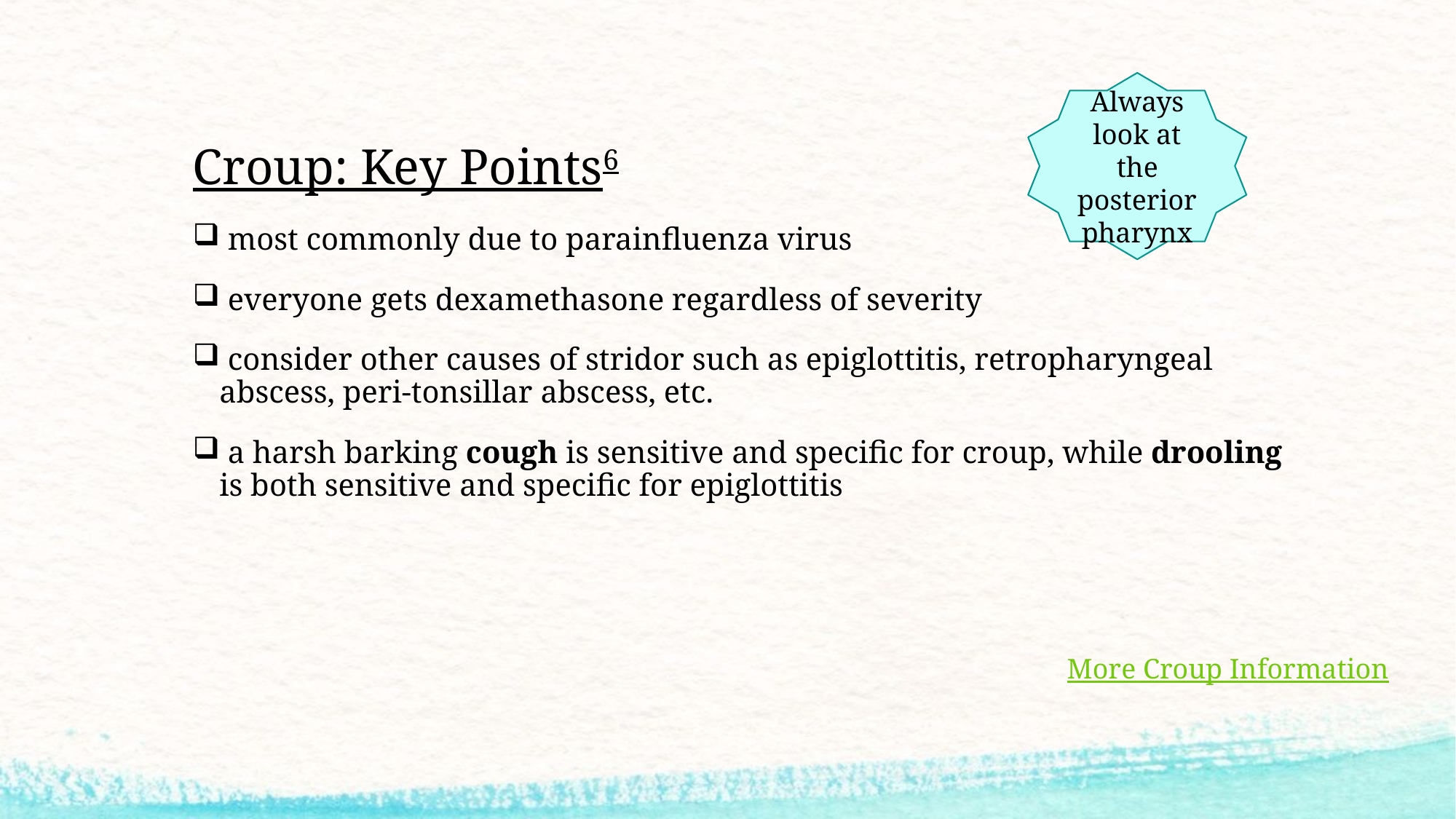

# Croup: Key Points6
Always look at the posterior pharynx
 most commonly due to parainfluenza virus
 everyone gets dexamethasone regardless of severity
 consider other causes of stridor such as epiglottitis, retropharyngeal abscess, peri-tonsillar abscess, etc.
 a harsh barking cough is sensitive and specific for croup, while drooling is both sensitive and specific for epiglottitis
More Croup Information

## Slide 20
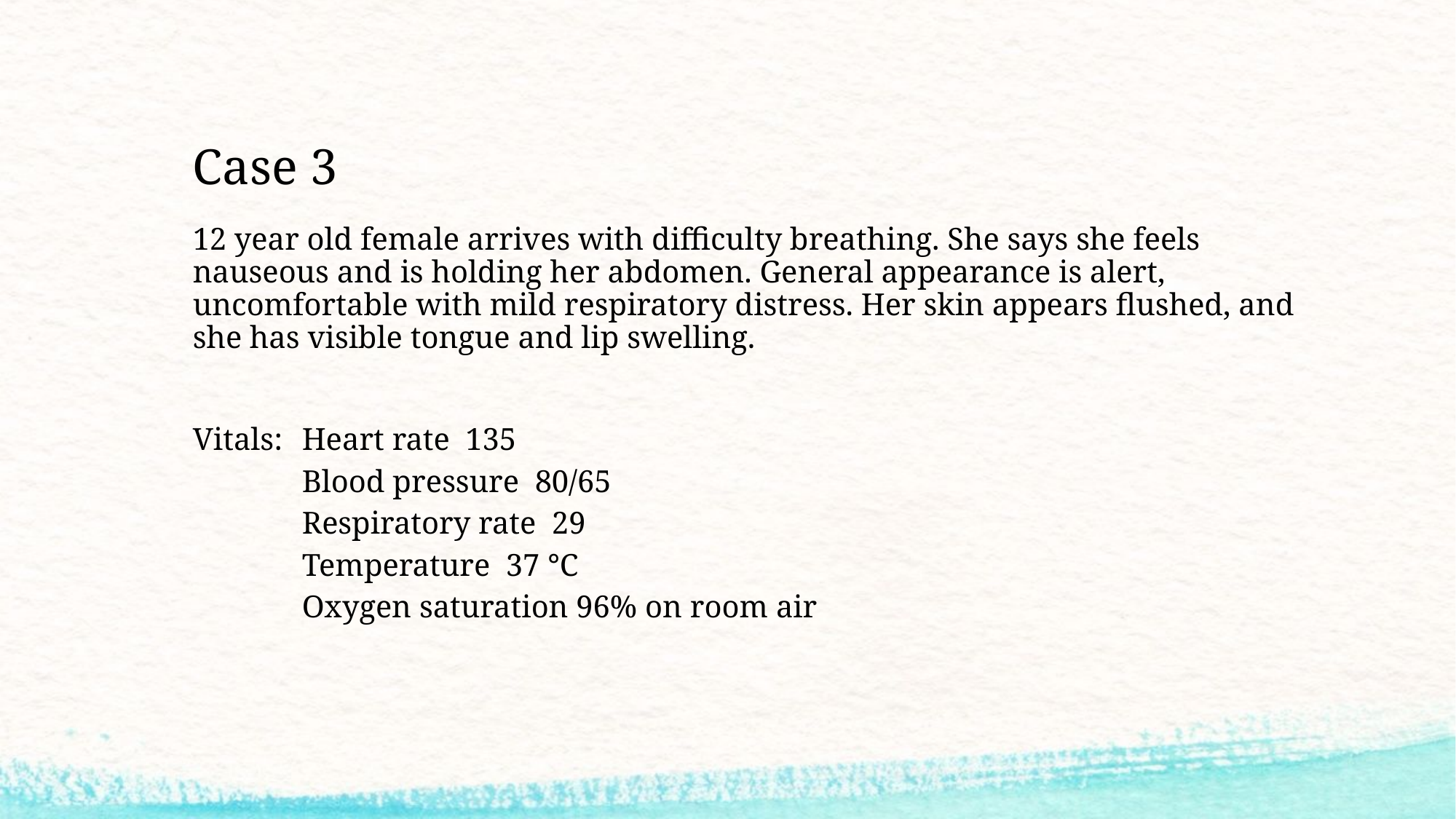

# Case 3
12 year old female arrives with difficulty breathing. She says she feels nauseous and is holding her abdomen. General appearance is alert, uncomfortable with mild respiratory distress. Her skin appears flushed, and she has visible tongue and lip swelling.
Vitals: 	Heart rate 135
	Blood pressure 80/65
	Respiratory rate 29
	Temperature 37 °C
	Oxygen saturation 96% on room air

## Slide 21
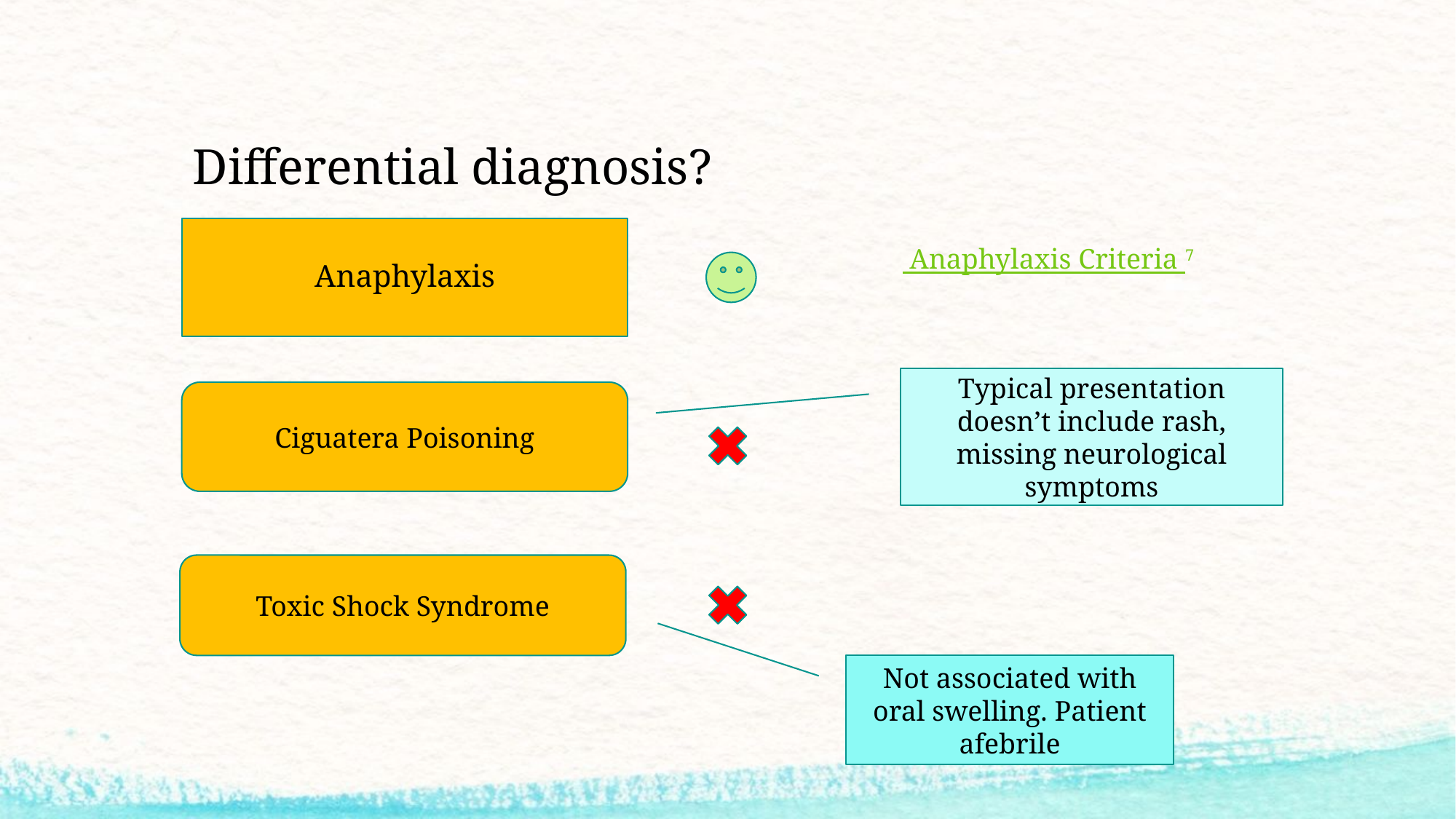

# Differential diagnosis?
Anaphylaxis
 Anaphylaxis Criteria 7
Typical presentation doesn’t include rash, missing neurological symptoms
Ciguatera Poisoning
Toxic Shock Syndrome
Not associated with oral swelling. Patient afebrile

## Slide 22
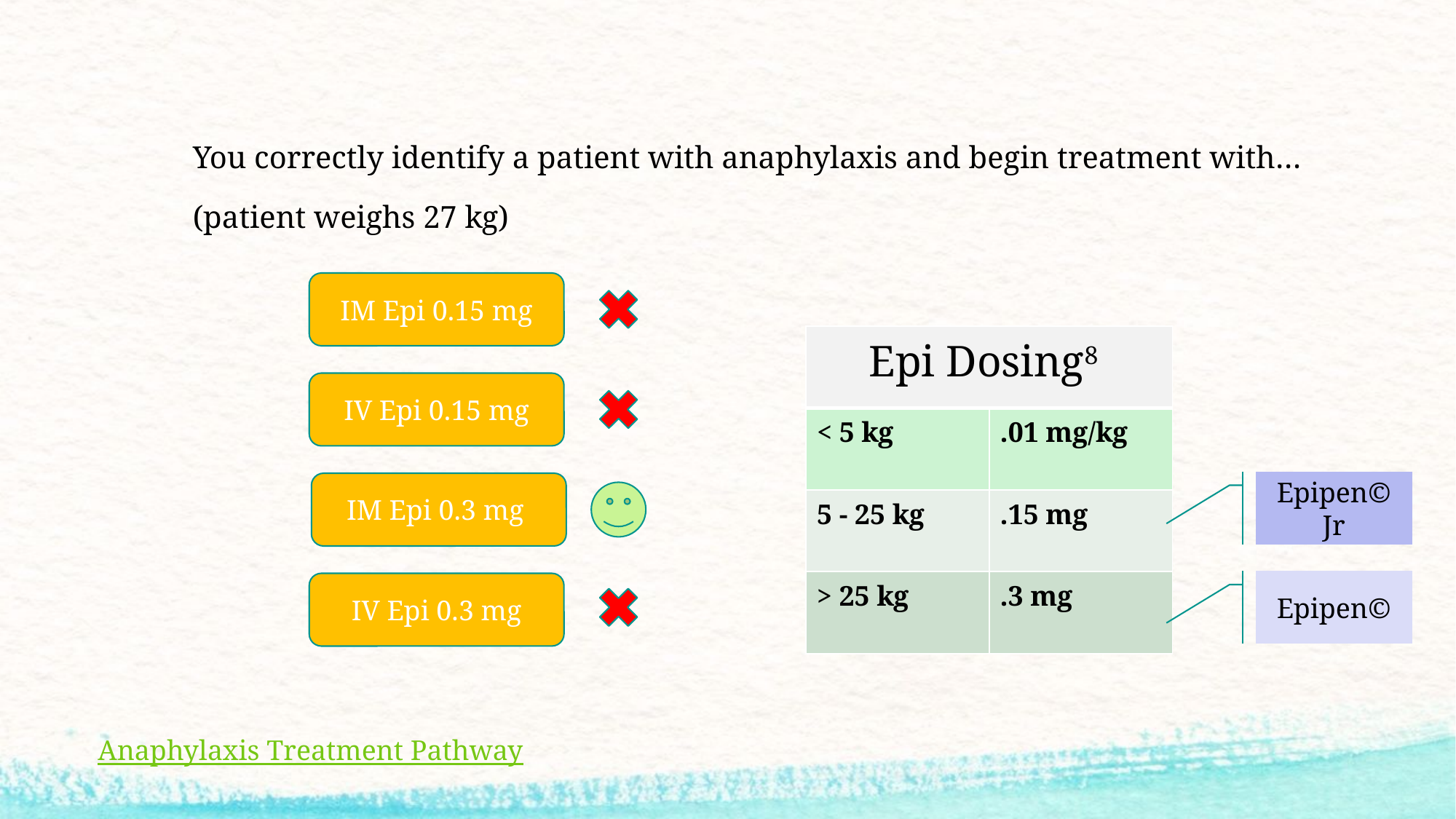

You correctly identify a patient with anaphylaxis and begin treatment with…
(patient weighs 27 kg)
IM Epi 0.15 mg
| Epi Dosing8 | |
| --- | --- |
| < 5 kg | .01 mg/kg |
| 5 - 25 kg | .15 mg |
| > 25 kg | .3 mg |
IV Epi 0.15 mg
Epipen© Jr
IM Epi 0.3 mg
Epipen©
IV Epi 0.3 mg
Anaphylaxis Treatment Pathway

## Slide 23
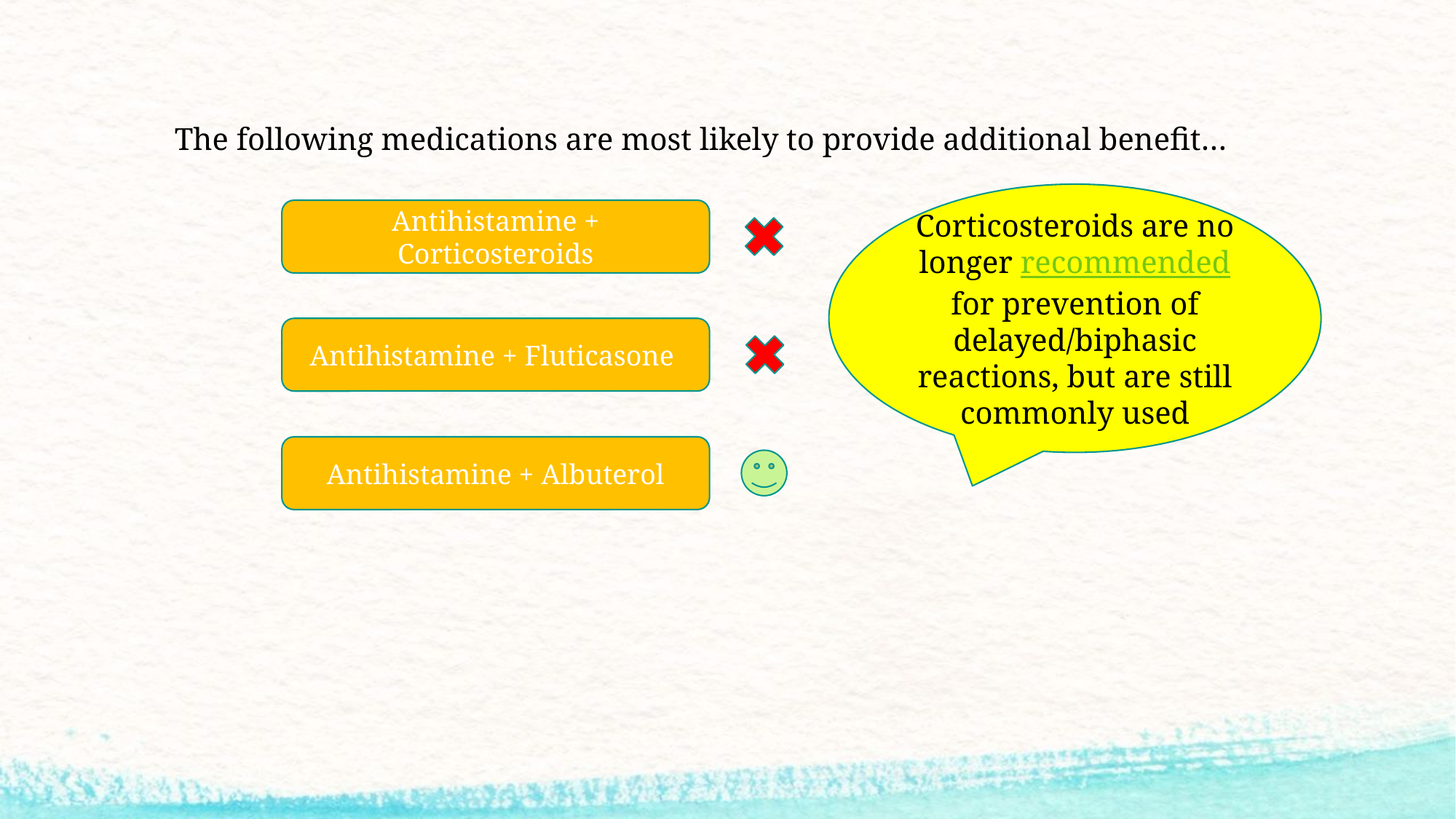

The following medications are most likely to provide additional benefit…
Corticosteroids are no longer recommended for prevention of delayed/biphasic reactions, but are still commonly used
Antihistamine + Corticosteroids
Antihistamine + Fluticasone
Antihistamine + Albuterol

## Slide 24
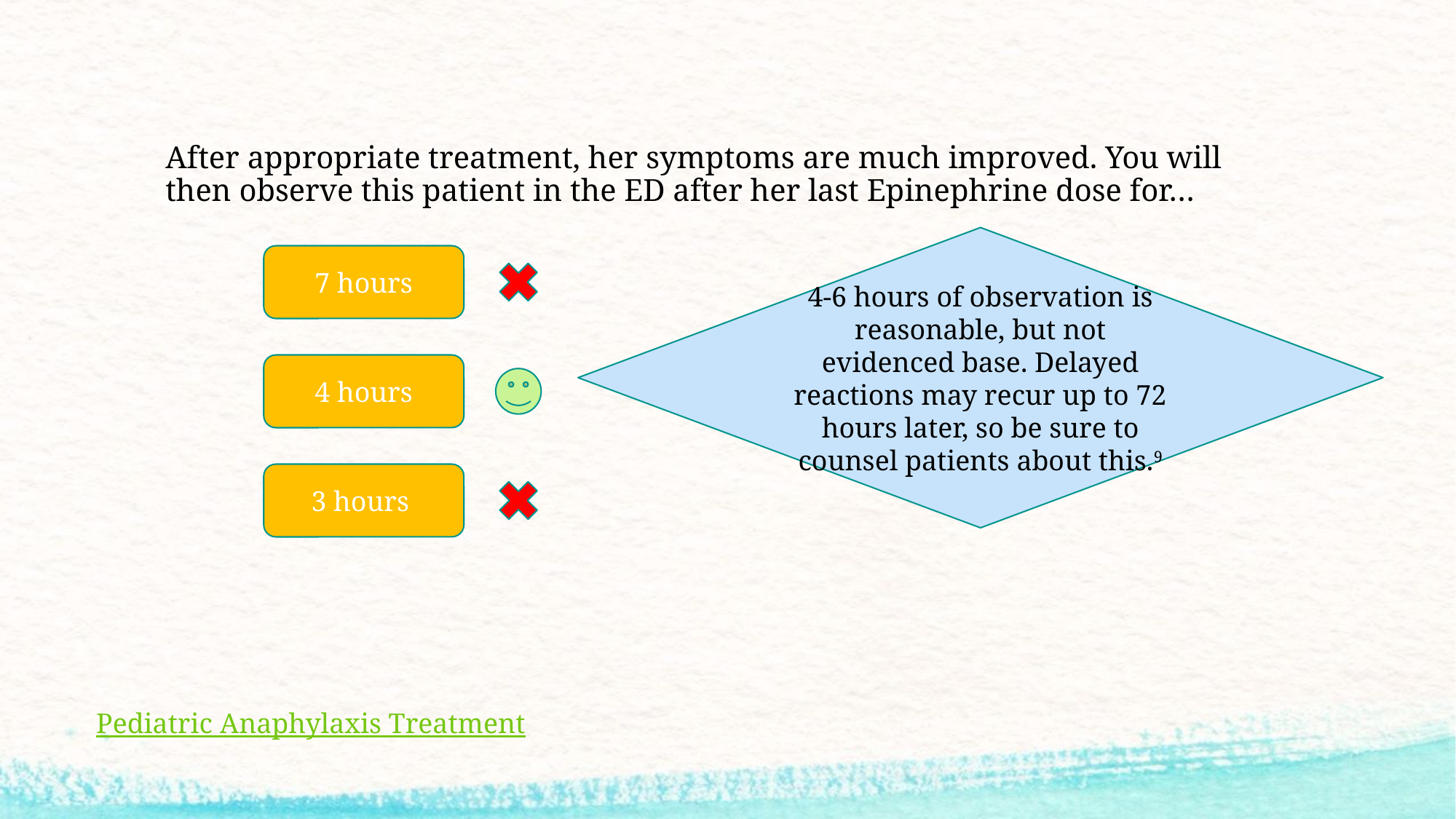

After appropriate treatment, her symptoms are much improved. You will then observe this patient in the ED after her last Epinephrine dose for…
4-6 hours of observation is reasonable, but not evidenced base. Delayed reactions may recur up to 72 hours later, so be sure to counsel patients about this.9
7 hours
4 hours
3 hours
Pediatric Anaphylaxis Treatment

## Slide 25
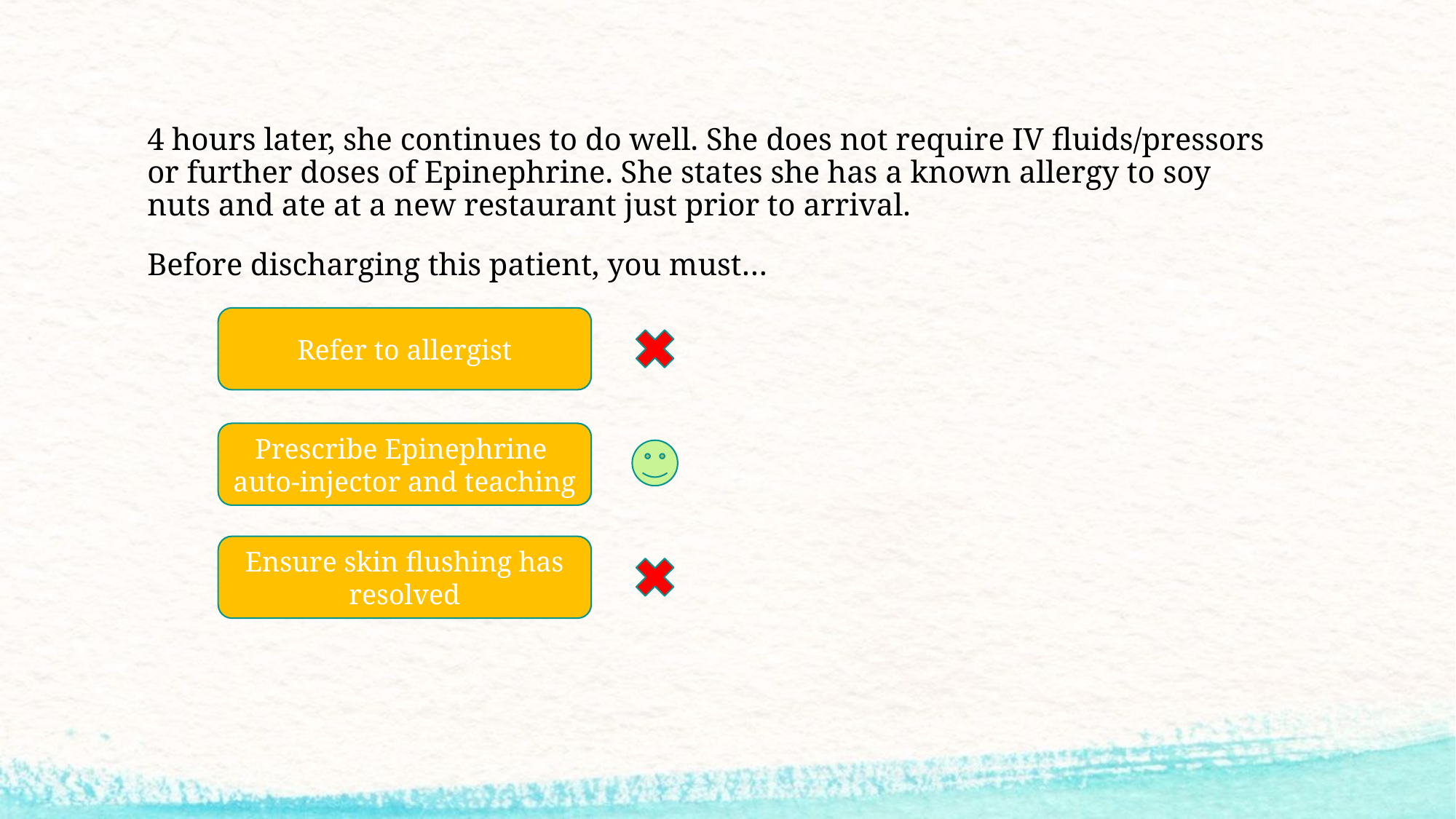

4 hours later, she continues to do well. She does not require IV fluids/pressors or further doses of Epinephrine. She states she has a known allergy to soy nuts and ate at a new restaurant just prior to arrival.
Before discharging this patient, you must…
Refer to allergist
Prescribe Epinephrine
auto-injector and teaching
Ensure skin flushing has resolved

## Slide 26
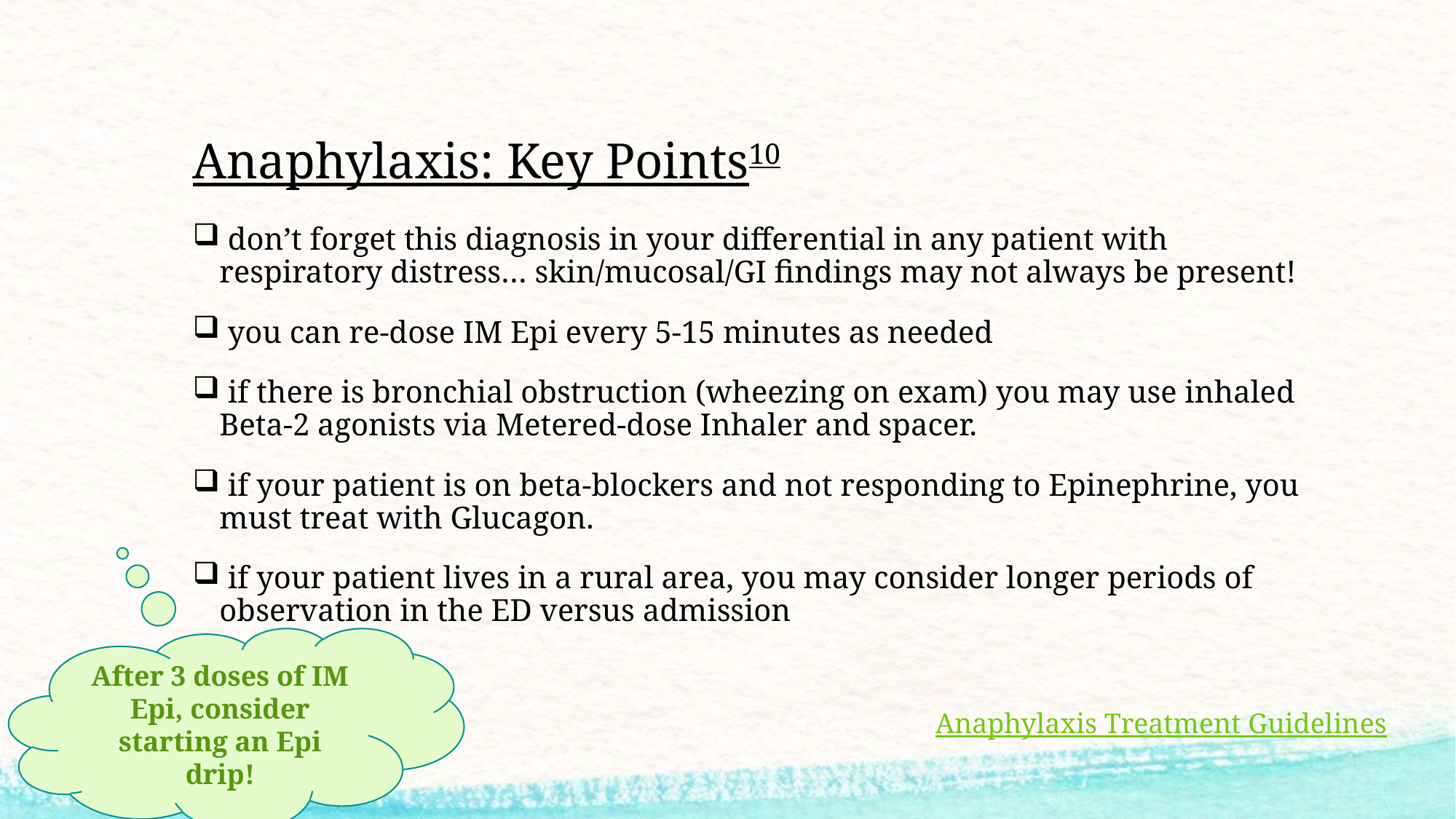

# Anaphylaxis: Key Points10
 don’t forget this diagnosis in your differential in any patient with respiratory distress… skin/mucosal/GI findings may not always be present!
 you can re-dose IM Epi every 5-15 minutes as needed
 if there is bronchial obstruction (wheezing on exam) you may use inhaled Beta-2 agonists via Metered-dose Inhaler and spacer.
 if your patient is on beta-blockers and not responding to Epinephrine, you must treat with Glucagon.
 if your patient lives in a rural area, you may consider longer periods of observation in the ED versus admission
After 3 doses of IM Epi, consider starting an Epi drip!
Anaphylaxis Treatment Guidelines

## Slide 27
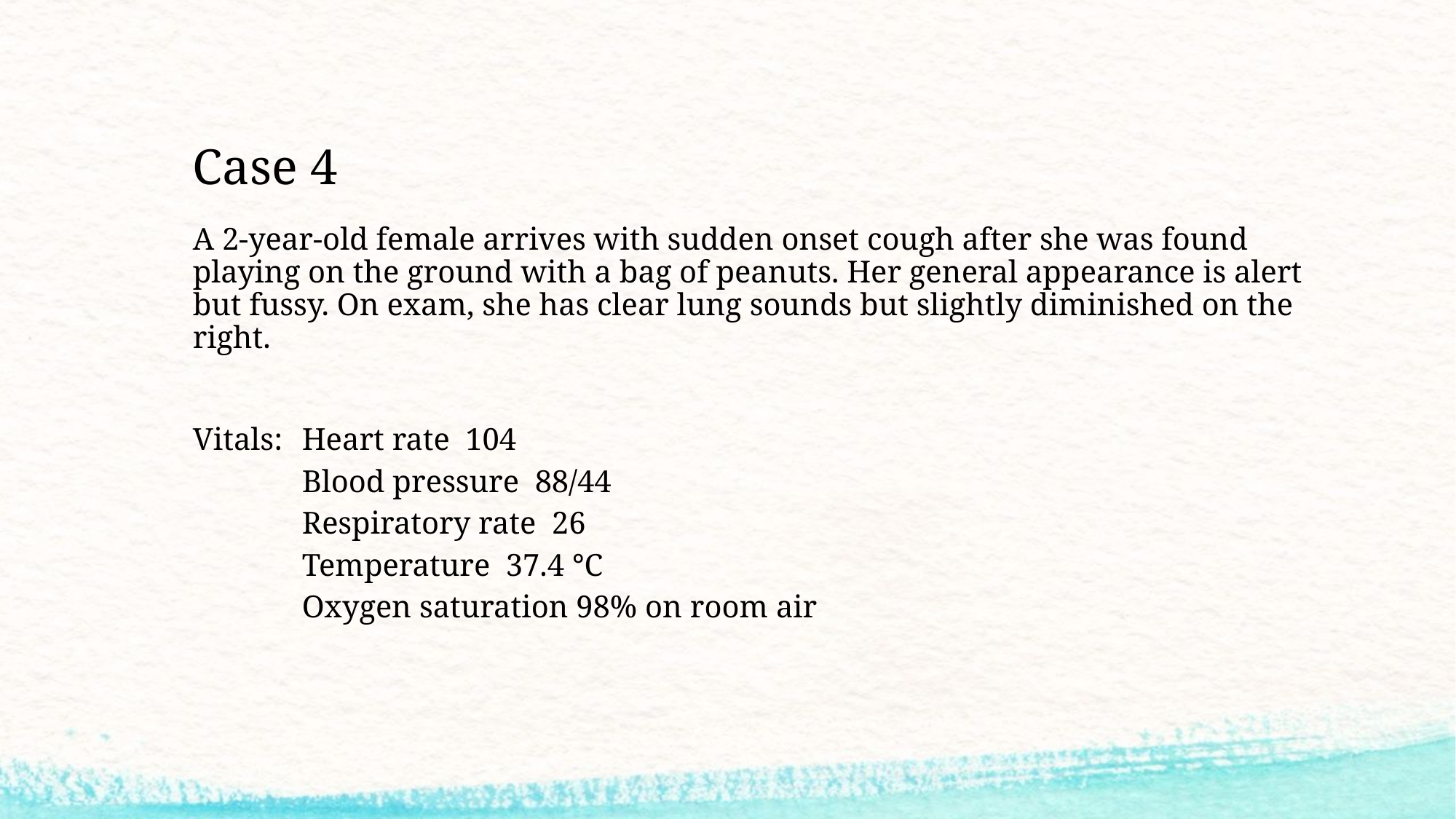

# Case 4
A 2-year-old female arrives with sudden onset cough after she was found playing on the ground with a bag of peanuts. Her general appearance is alert but fussy. On exam, she has clear lung sounds but slightly diminished on the right.
Vitals: 	Heart rate 104
	Blood pressure 88/44
	Respiratory rate 26
	Temperature 37.4 °C
	Oxygen saturation 98% on room air

## Slide 28
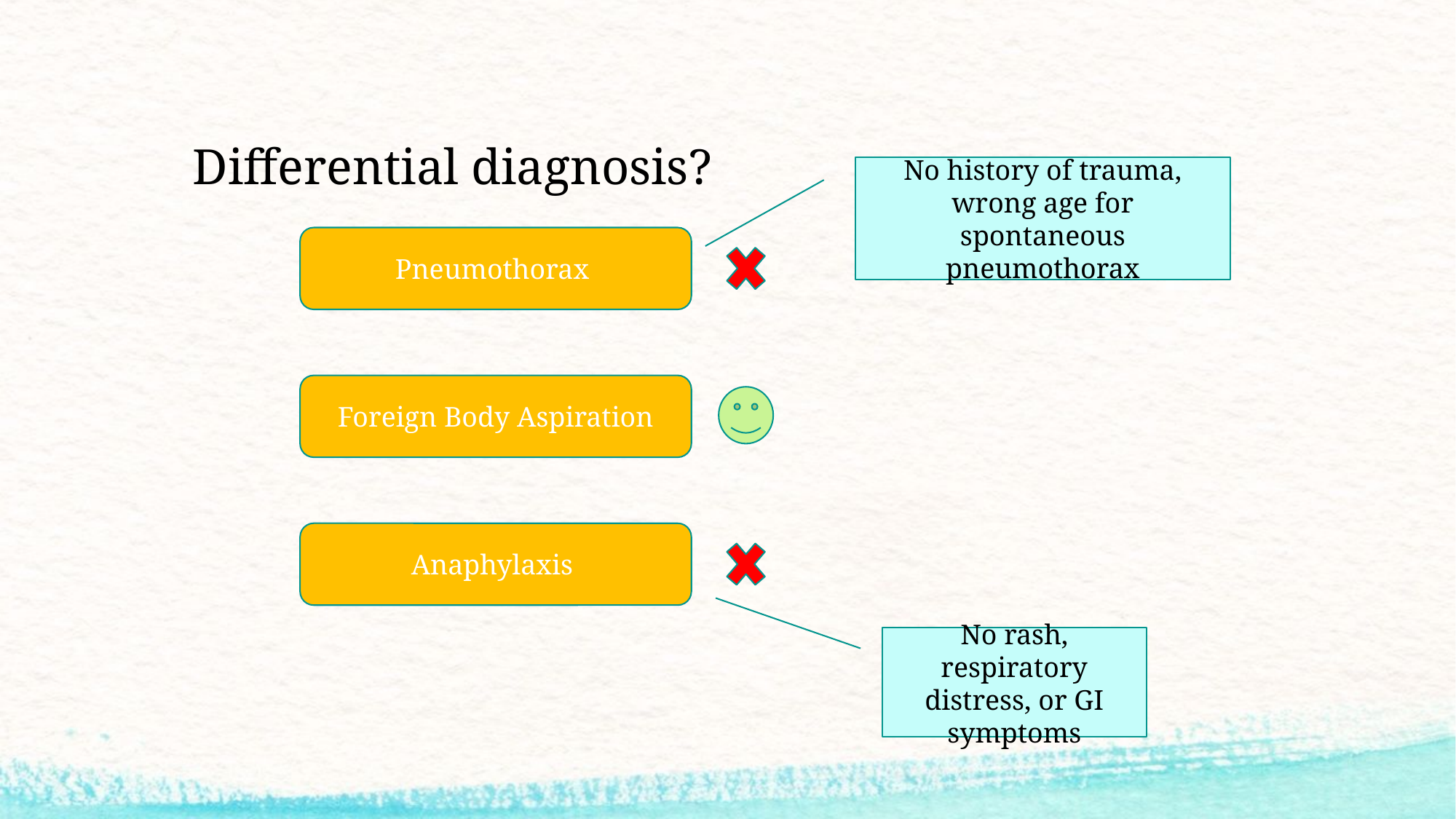

# Differential diagnosis?
No history of trauma, wrong age for spontaneous pneumothorax
Pneumothorax
Foreign Body Aspiration
Anaphylaxis
No rash, respiratory distress, or GI symptoms

## Slide 29
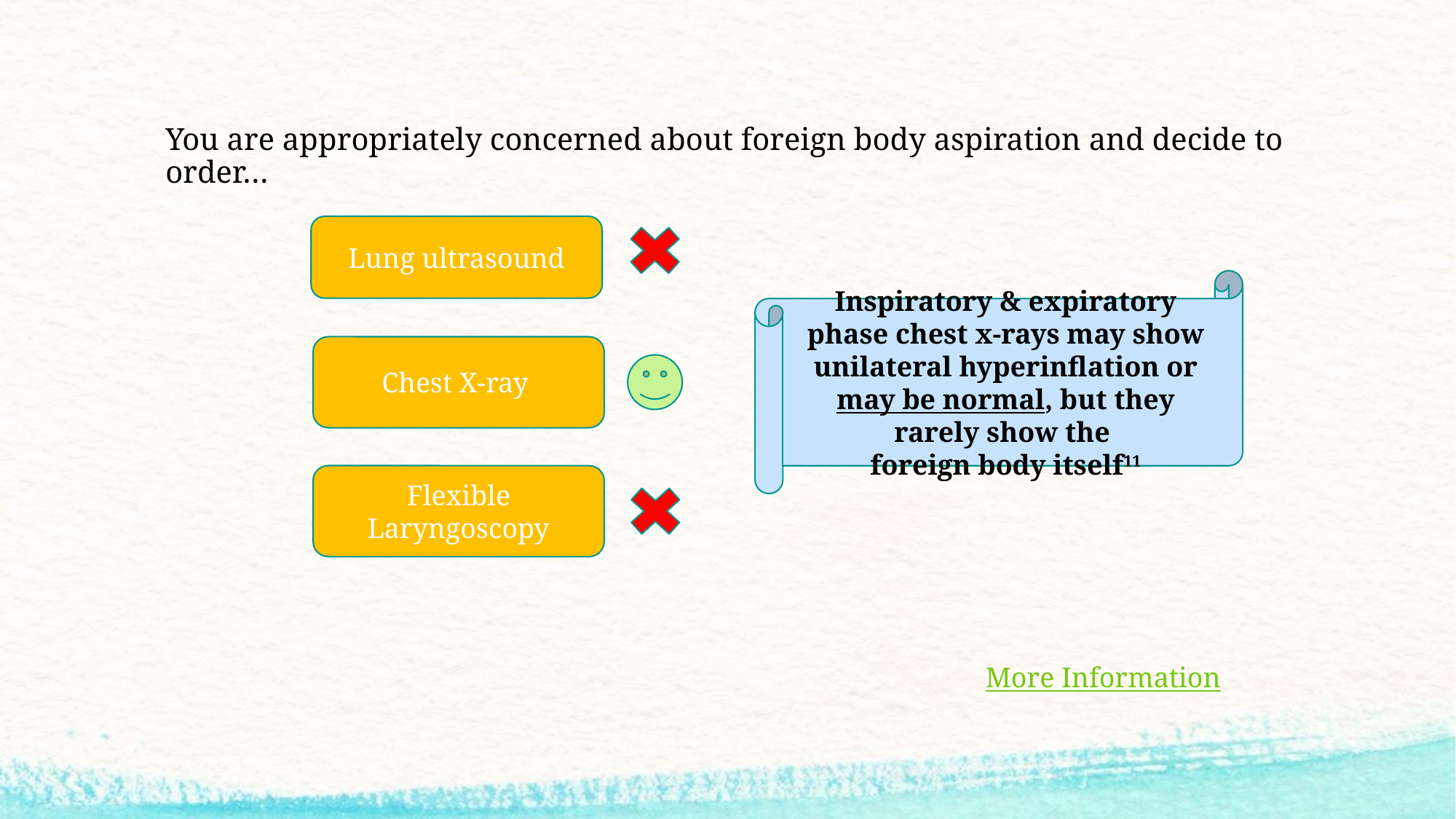

You are appropriately concerned about foreign body aspiration and decide to order…
Lung ultrasound
Inspiratory & expiratory phase chest x-rays may show unilateral hyperinflation or may be normal, but they rarely show the
foreign body itself11
Chest X-ray
Flexible Laryngoscopy
More Information

## Slide 30
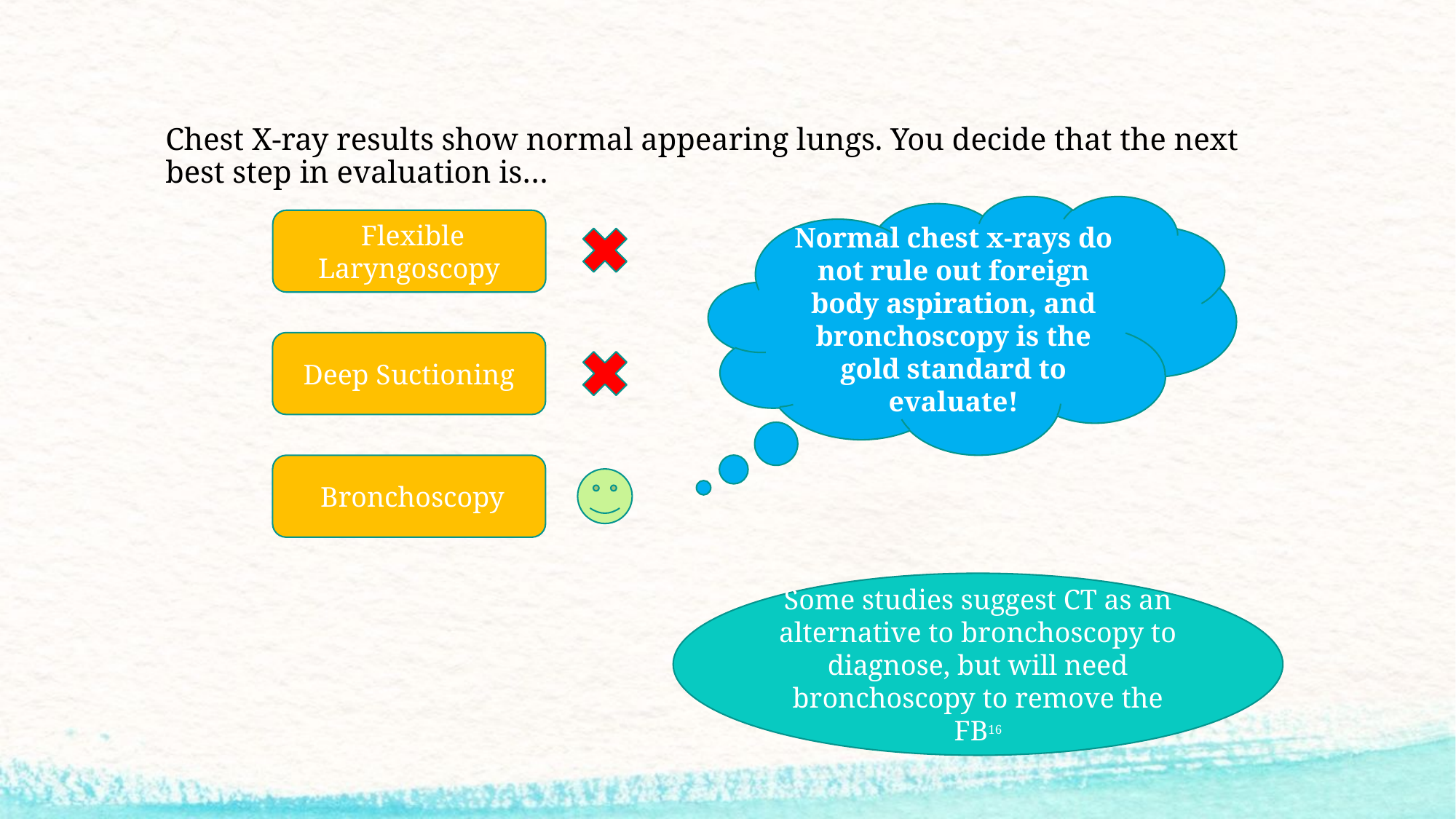

Chest X-ray results show normal appearing lungs. You decide that the next best step in evaluation is…
Normal chest x-rays do not rule out foreign body aspiration, and bronchoscopy is the gold standard to evaluate!
 Flexible Laryngoscopy
 Deep Suctioning
 Bronchoscopy
Some studies suggest CT as an alternative to bronchoscopy to diagnose, but will need bronchoscopy to remove the FB16

## Slide 31
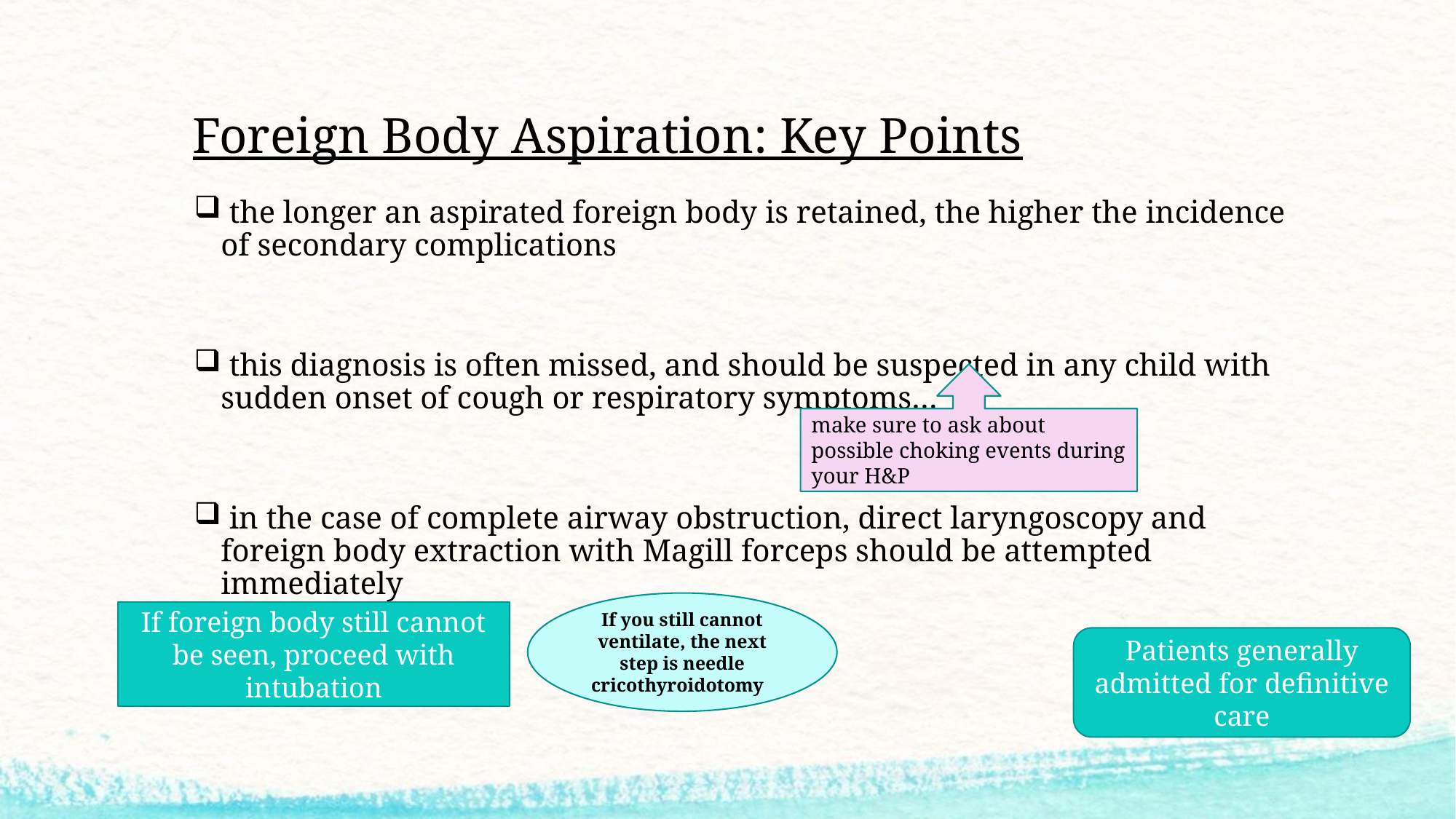

# Foreign Body Aspiration: Key Points
 the longer an aspirated foreign body is retained, the higher the incidence of secondary complications
 this diagnosis is often missed, and should be suspected in any child with sudden onset of cough or respiratory symptoms…
 in the case of complete airway obstruction, direct laryngoscopy and foreign body extraction with Magill forceps should be attempted immediately
make sure to ask about possible choking events during your H&P
If you still cannot ventilate, the next step is needle cricothyroidotomy
If foreign body still cannot be seen, proceed with intubation
Patients generally admitted for definitive care

## Slide 32
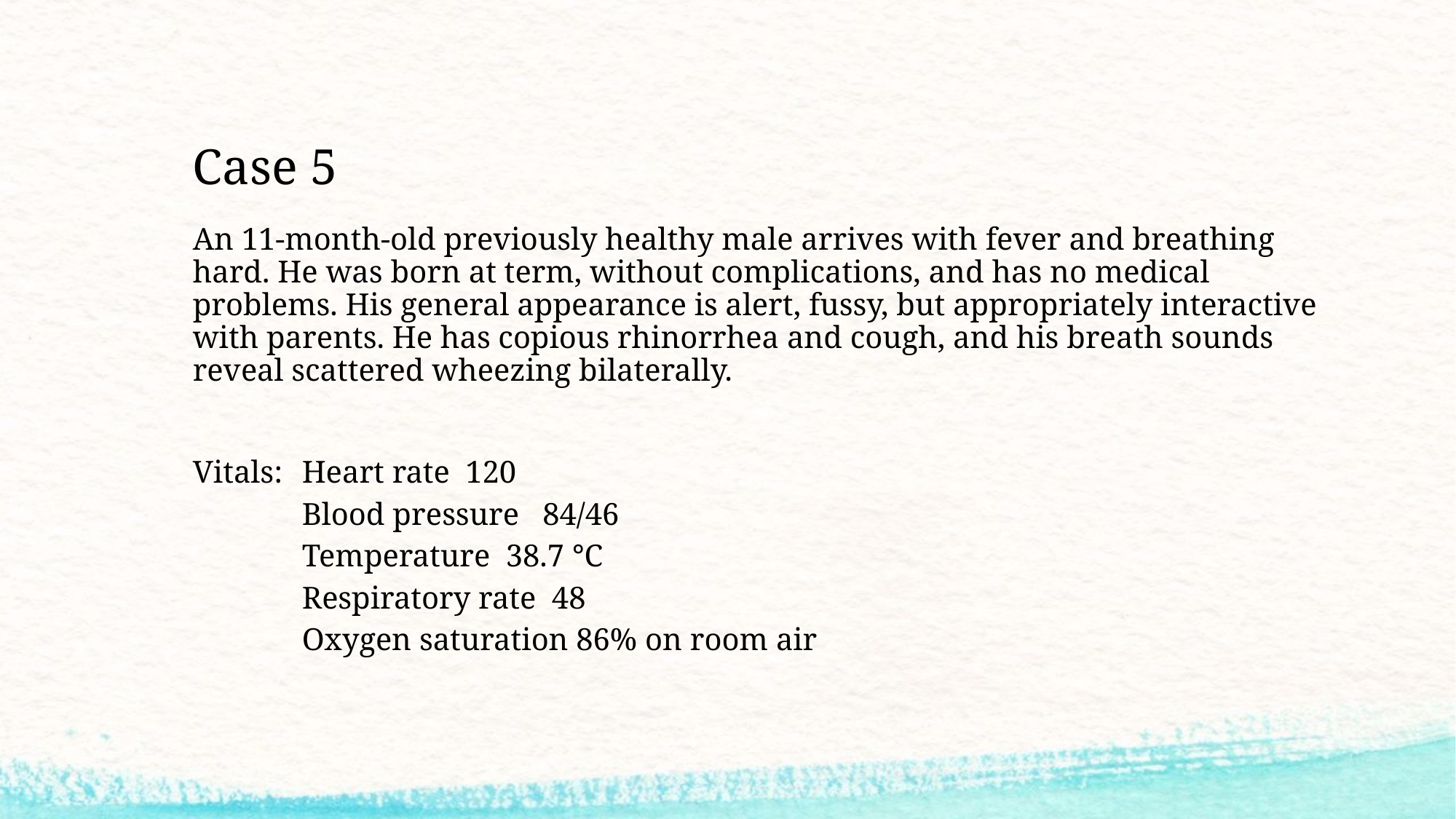

# Case 5
An 11-month-old previously healthy male arrives with fever and breathing hard. He was born at term, without complications, and has no medical problems. His general appearance is alert, fussy, but appropriately interactive with parents. He has copious rhinorrhea and cough, and his breath sounds reveal scattered wheezing bilaterally.
Vitals: 	Heart rate 120
	Blood pressure 84/46
	Temperature 38.7 °C
	Respiratory rate 48
	Oxygen saturation 86% on room air

## Slide 33
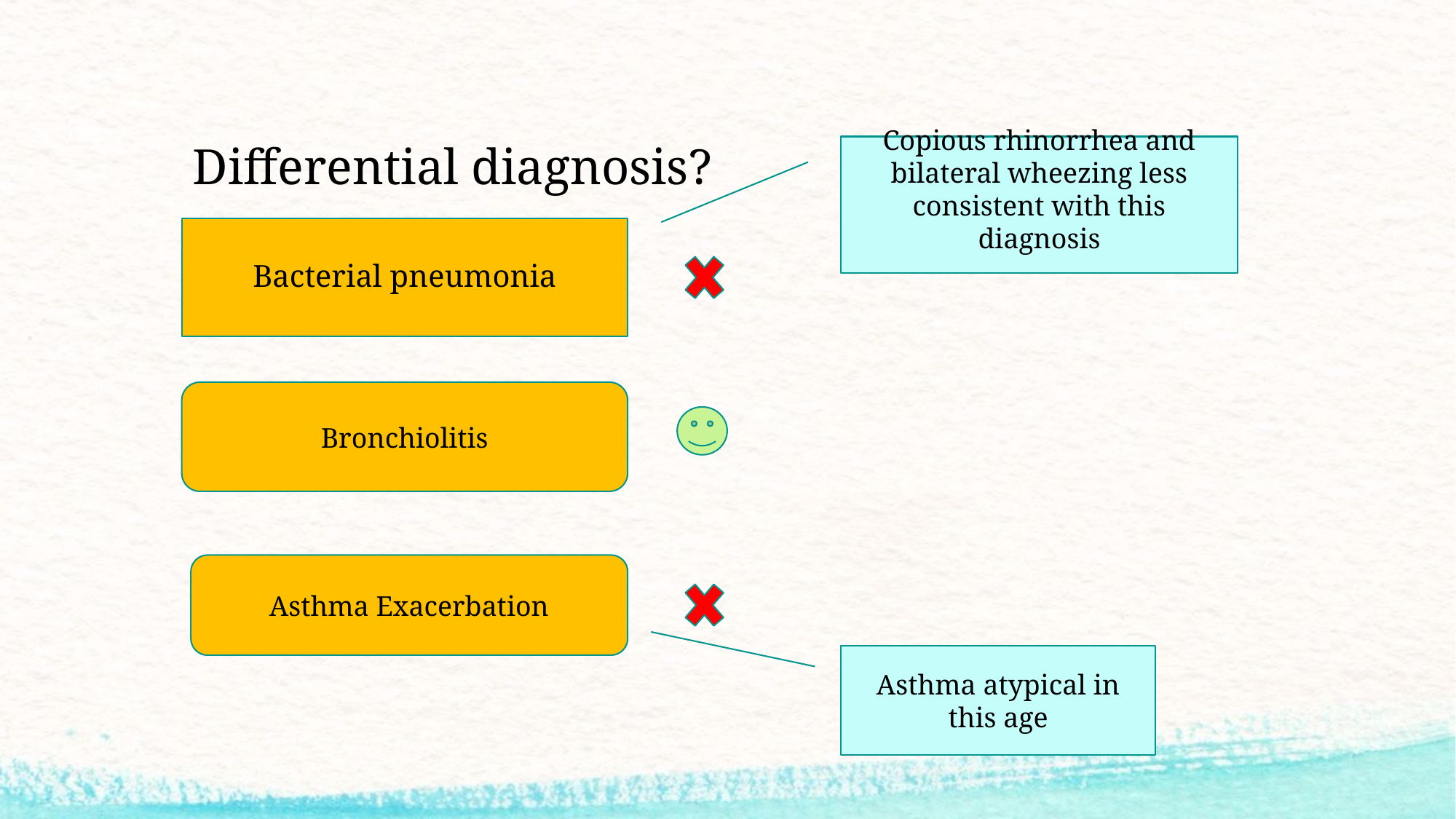

# Differential diagnosis?
Copious rhinorrhea and bilateral wheezing less consistent with this diagnosis
Bacterial pneumonia
Bronchiolitis
Asthma Exacerbation
Asthma atypical in this age

## Slide 34
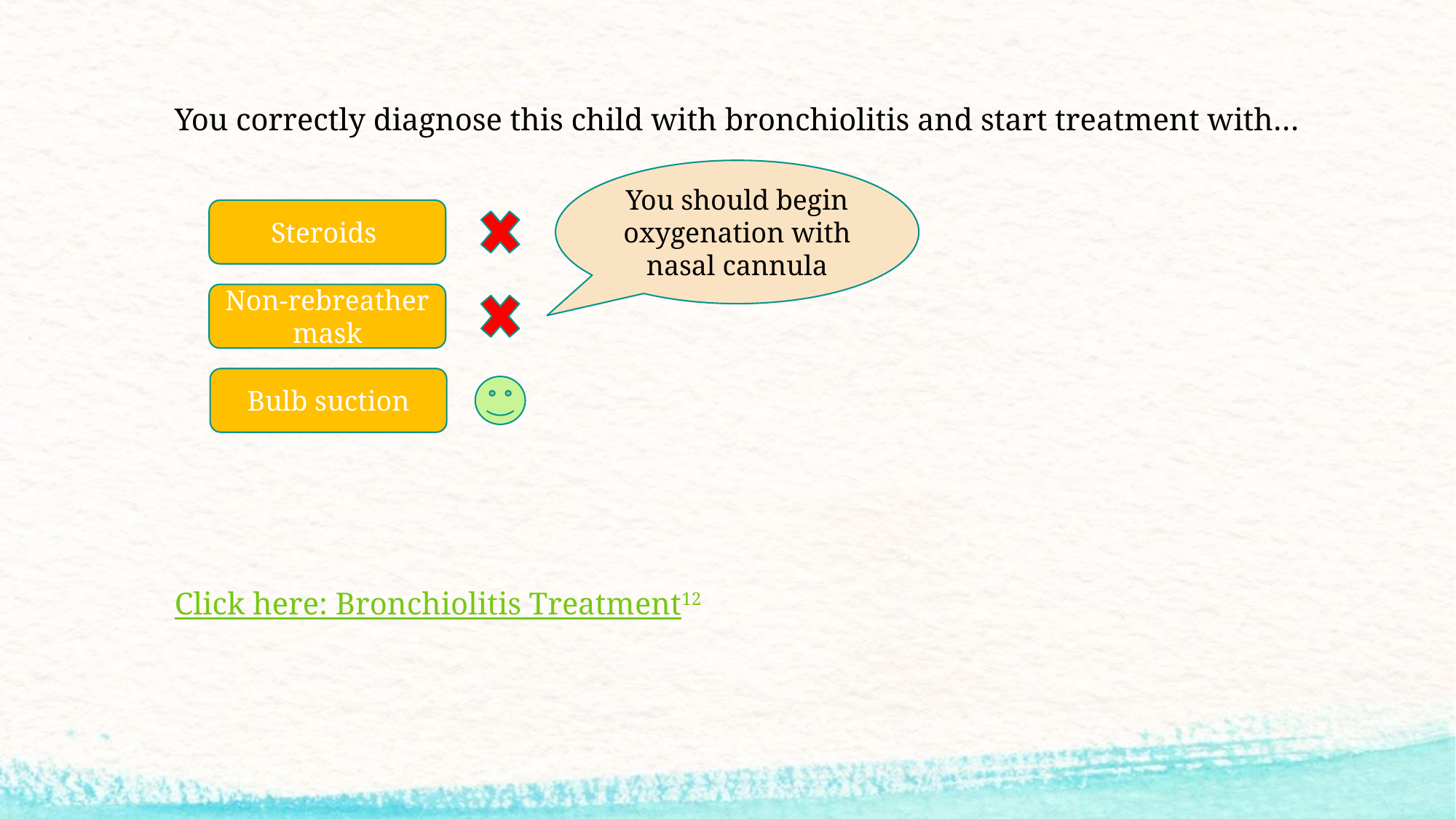

You correctly diagnose this child with bronchiolitis and start treatment with…
Click here: Bronchiolitis Treatment12
You should begin oxygenation with nasal cannula
Steroids
Non-rebreather mask
Bulb suction

## Slide 35
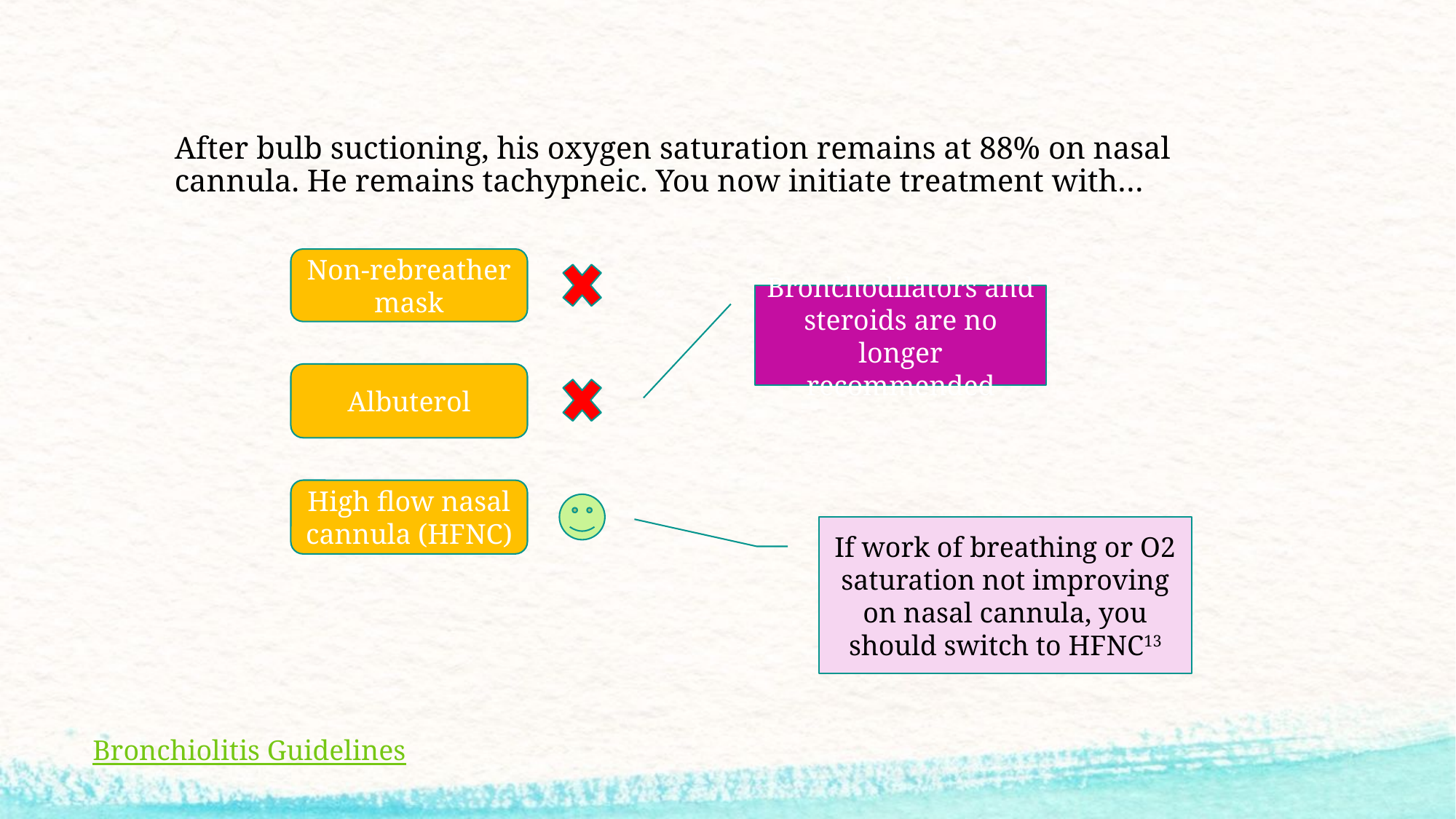

After bulb suctioning, his oxygen saturation remains at 88% on nasal cannula. He remains tachypneic. You now initiate treatment with…
Non-rebreather mask
Bronchodilators and steroids are no longer recommended
Albuterol
High flow nasal cannula (HFNC)
If work of breathing or O2 saturation not improving on nasal cannula, you should switch to HFNC13
Bronchiolitis Guidelines

## Slide 36
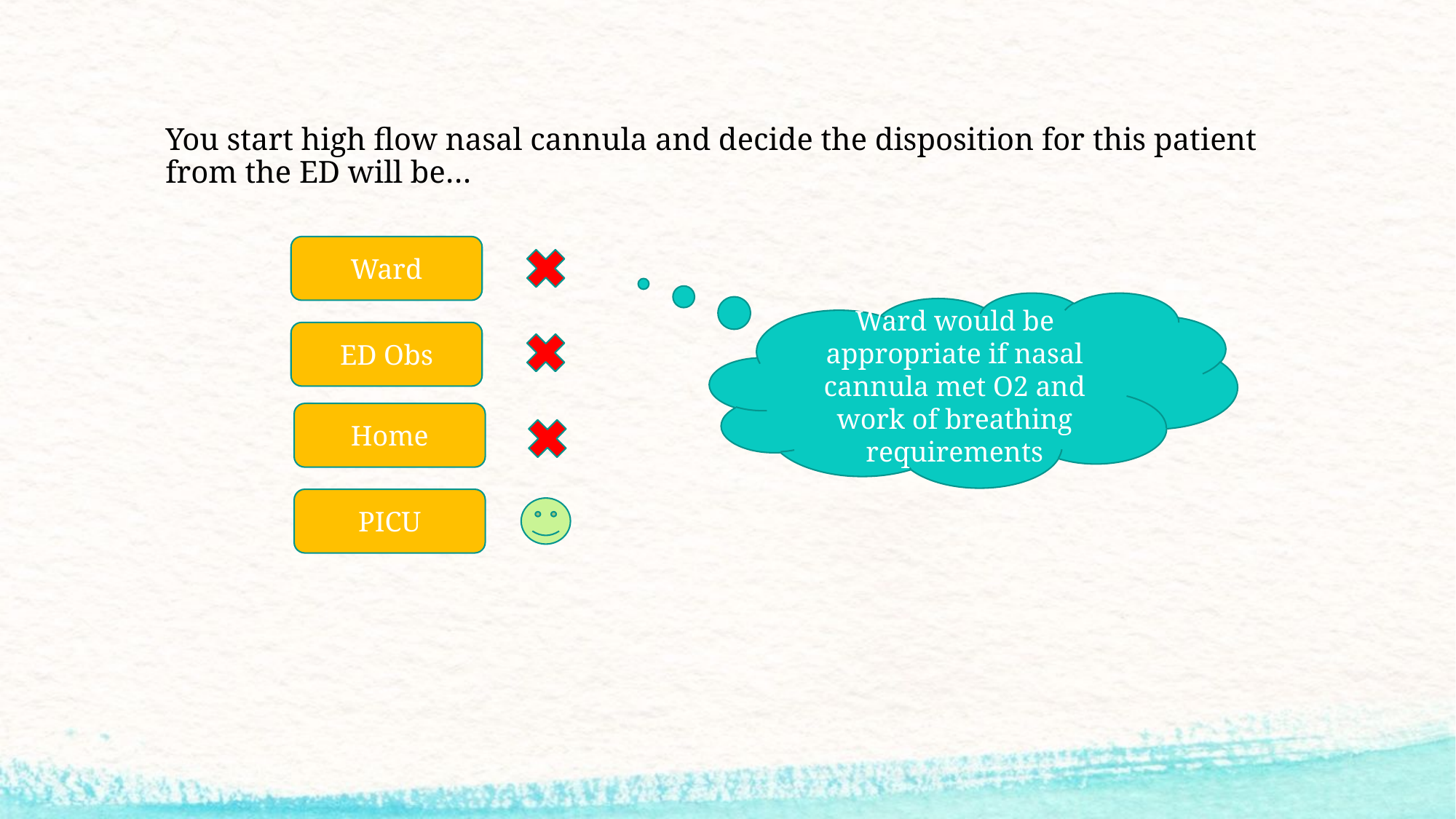

You start high flow nasal cannula and decide the disposition for this patient from the ED will be…
Ward
Ward would be appropriate if nasal cannula met O2 and work of breathing requirements
ED Obs
Home
PICU

## Slide 37
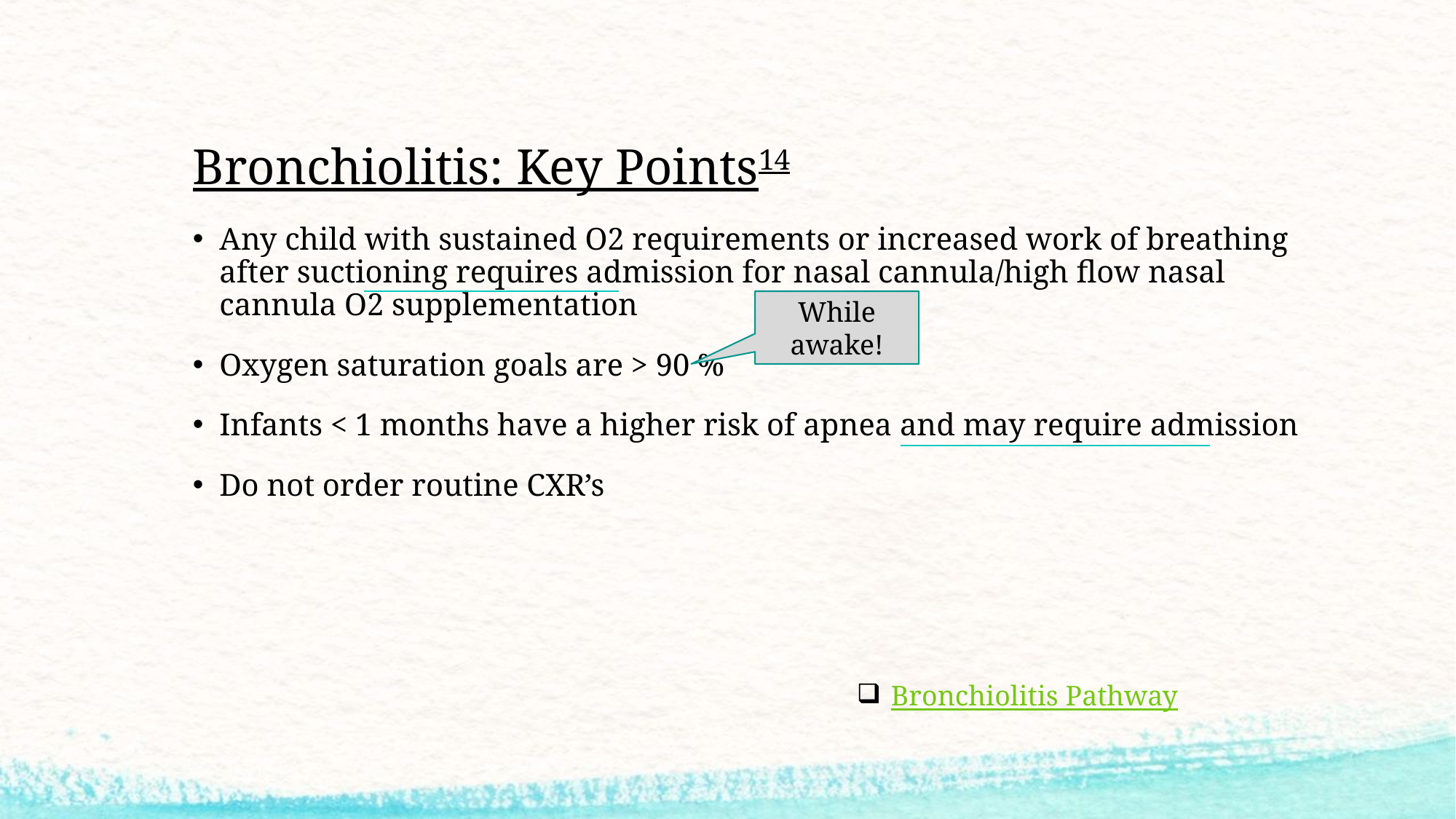

# Bronchiolitis: Key Points14
Any child with sustained O2 requirements or increased work of breathing after suctioning requires admission for nasal cannula/high flow nasal cannula O2 supplementation
Oxygen saturation goals are > 90 %
Infants < 1 months have a higher risk of apnea and may require admission
Do not order routine CXR’s
While awake!
Bronchiolitis Pathway

## Slide 38
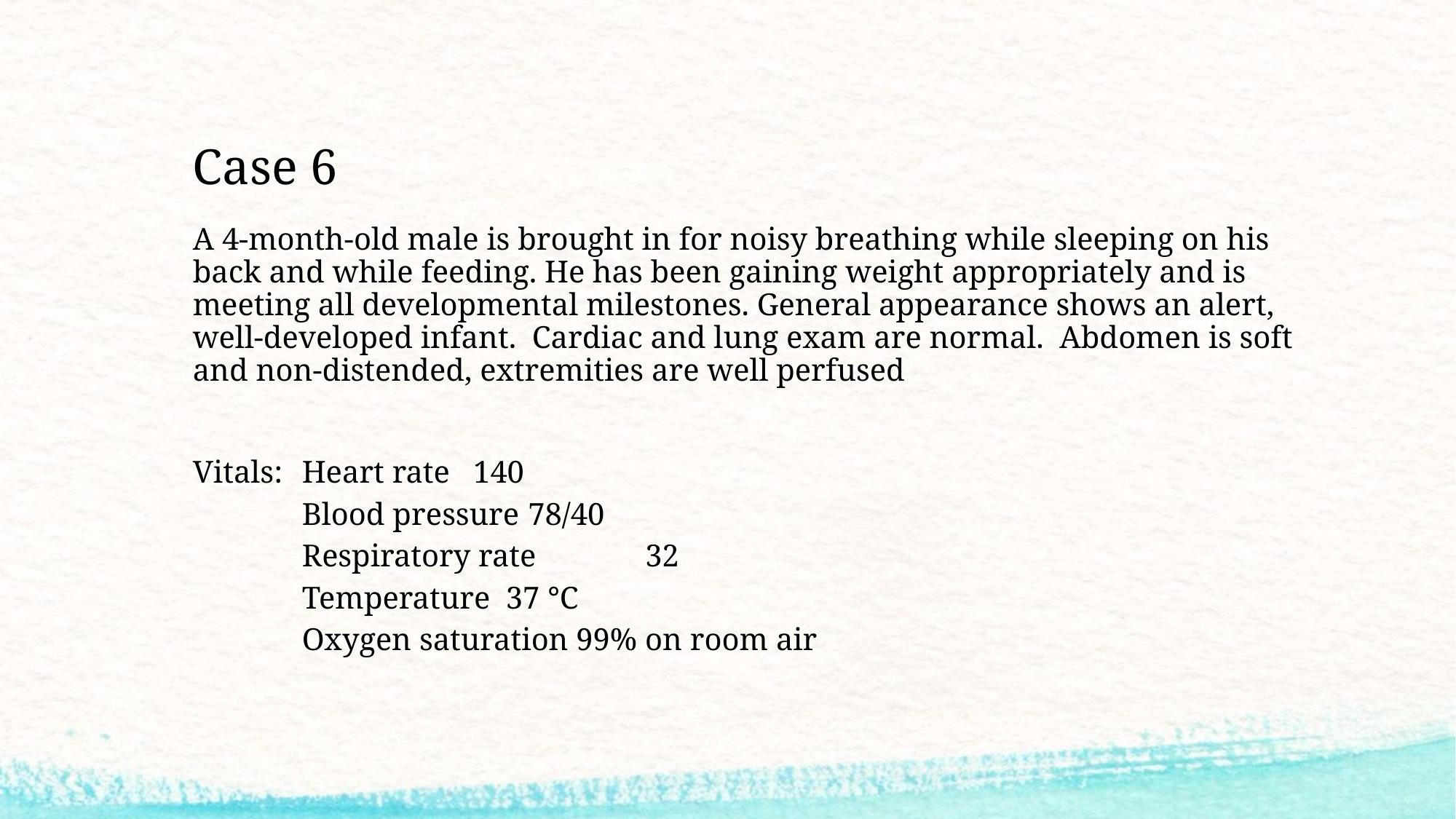

# Case 6
A 4-month-old male is brought in for noisy breathing while sleeping on his back and while feeding. He has been gaining weight appropriately and is meeting all developmental milestones. General appearance shows an alert, well-developed infant. Cardiac and lung exam are normal. Abdomen is soft and non-distended, extremities are well perfused
Vitals: 	Heart rate 140
	Blood pressure	 78/40
	Respiratory rate	 32
	Temperature 37 °C
	Oxygen saturation 99% on room air

## Slide 39
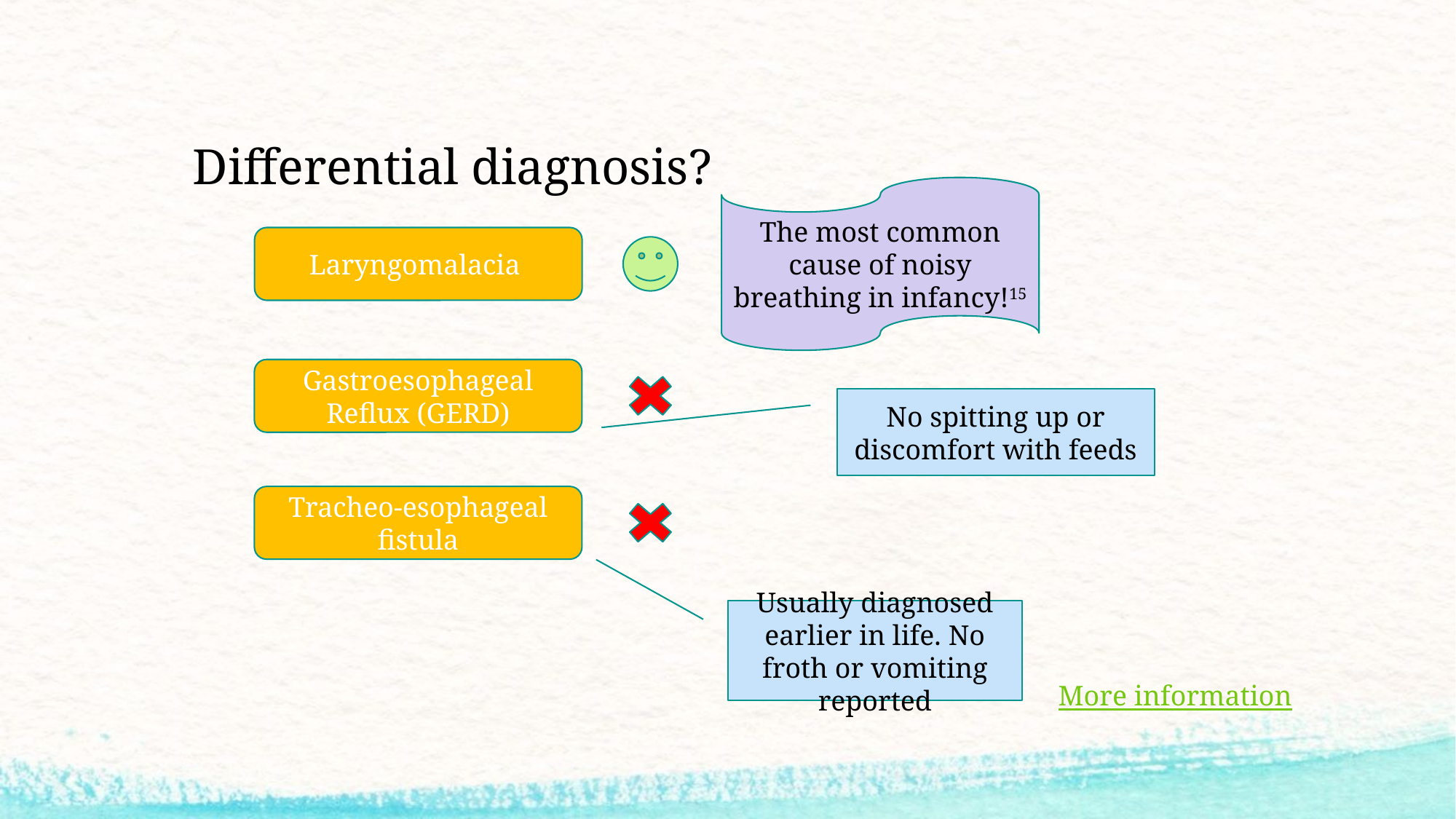

# Differential diagnosis?
The most common cause of noisy breathing in infancy!15
Laryngomalacia
Gastroesophageal Reflux (GERD)
No spitting up or discomfort with feeds
Tracheo-esophageal fistula
Usually diagnosed earlier in life. No froth or vomiting reported
More information

## Slide 40
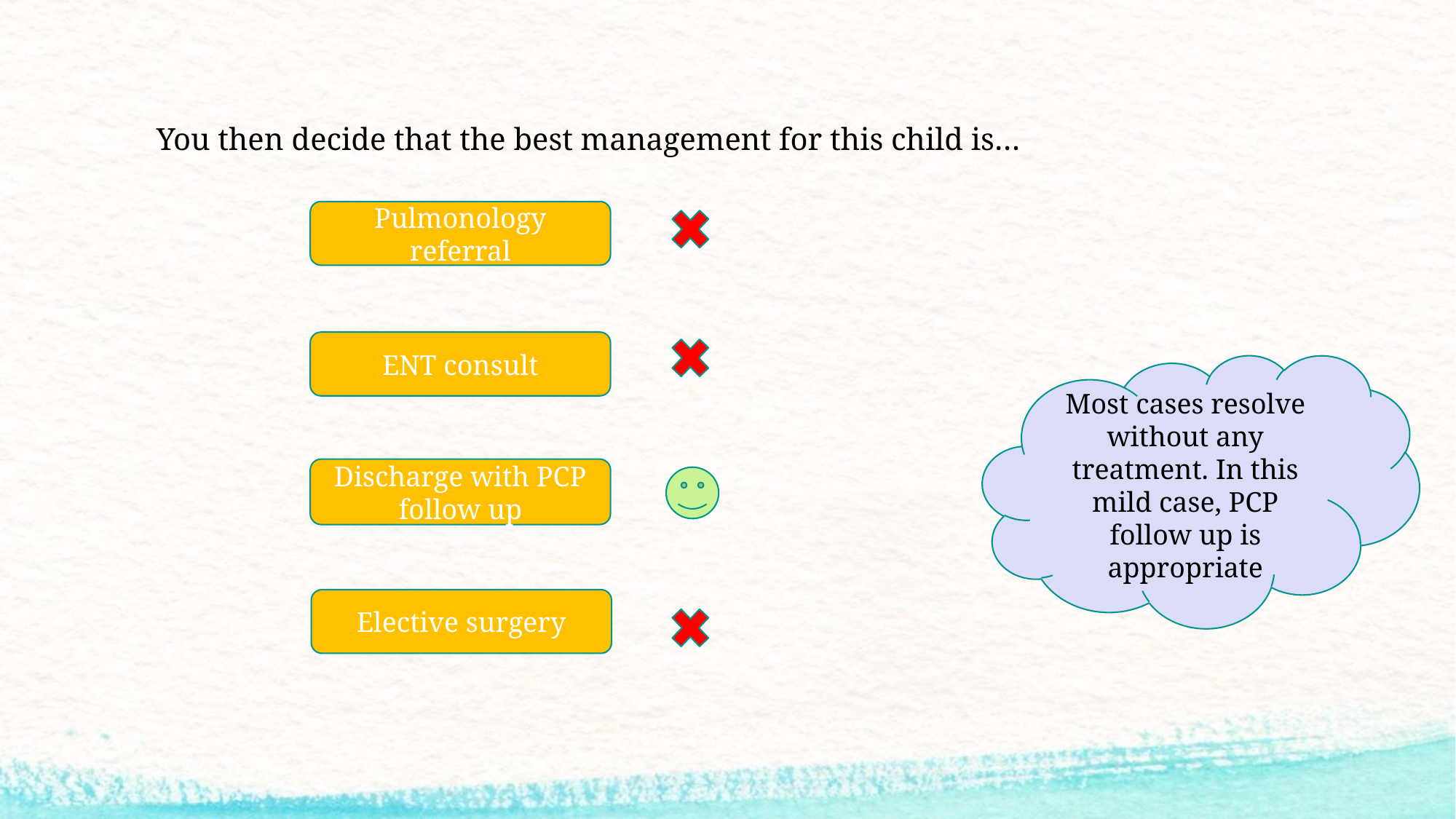

You then decide that the best management for this child is…
Pulmonology referral
ENT consult
Most cases resolve without any treatment. In this mild case, PCP follow up is appropriate
Discharge with PCP follow up
Elective surgery

## Slide 41
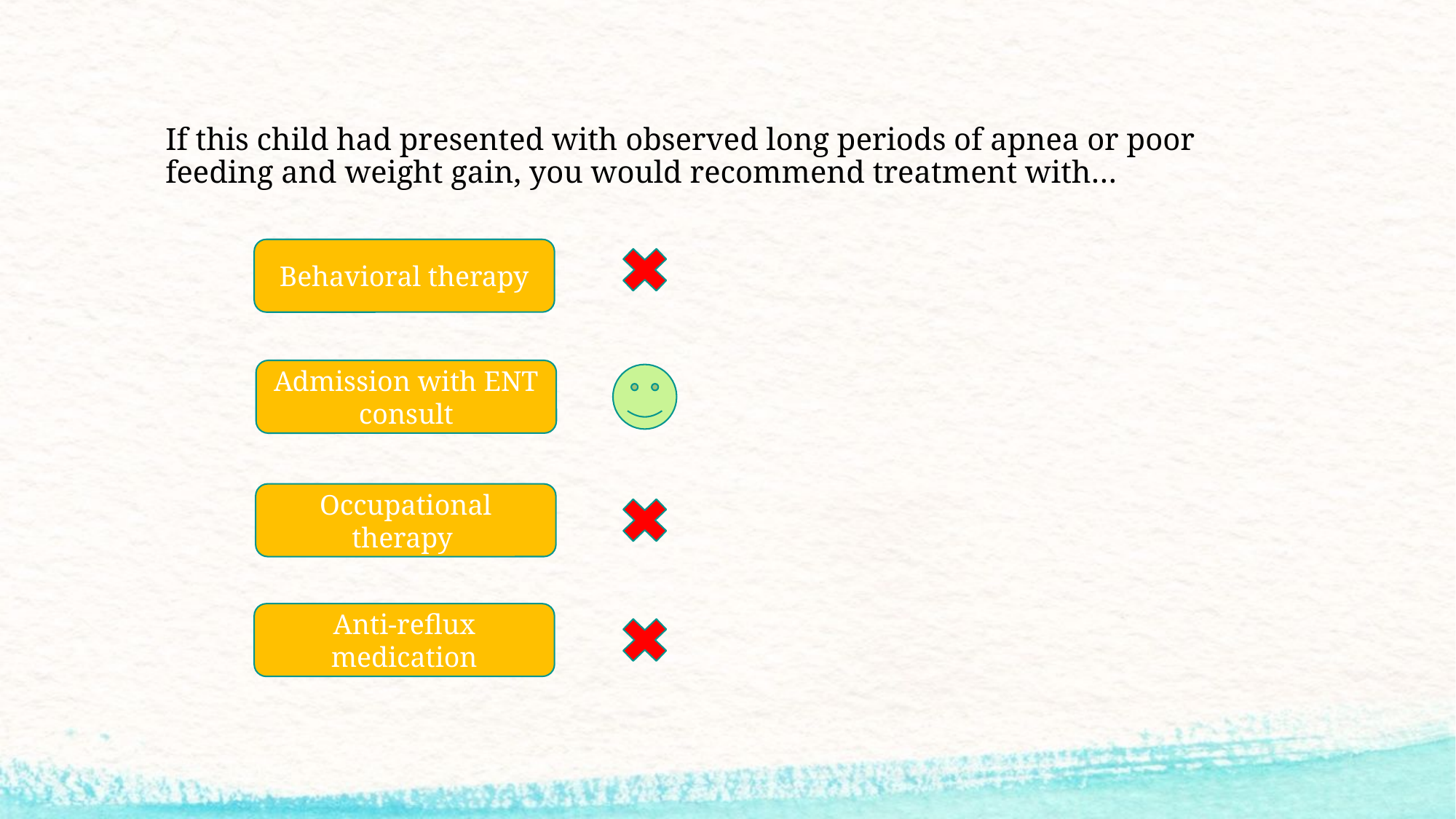

If this child had presented with observed long periods of apnea or poor feeding and weight gain, you would recommend treatment with…
Behavioral therapy
Admission with ENT consult
Occupational therapy
Anti-reflux medication

## Slide 42
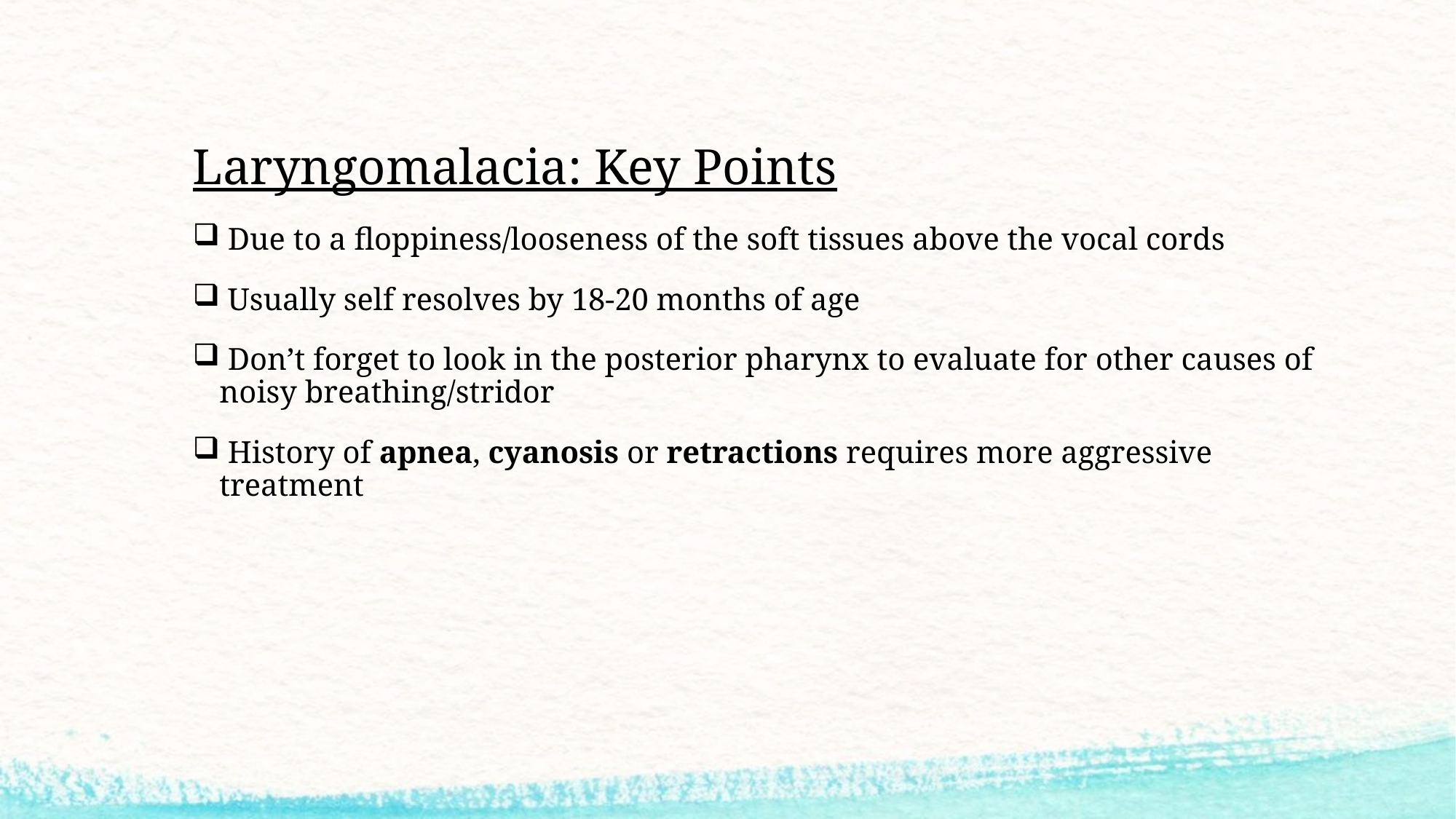

# Laryngomalacia: Key Points
 Due to a floppiness/looseness of the soft tissues above the vocal cords
 Usually self resolves by 18-20 months of age
 Don’t forget to look in the posterior pharynx to evaluate for other causes of noisy breathing/stridor
 History of apnea, cyanosis or retractions requires more aggressive treatment

## Slide 43
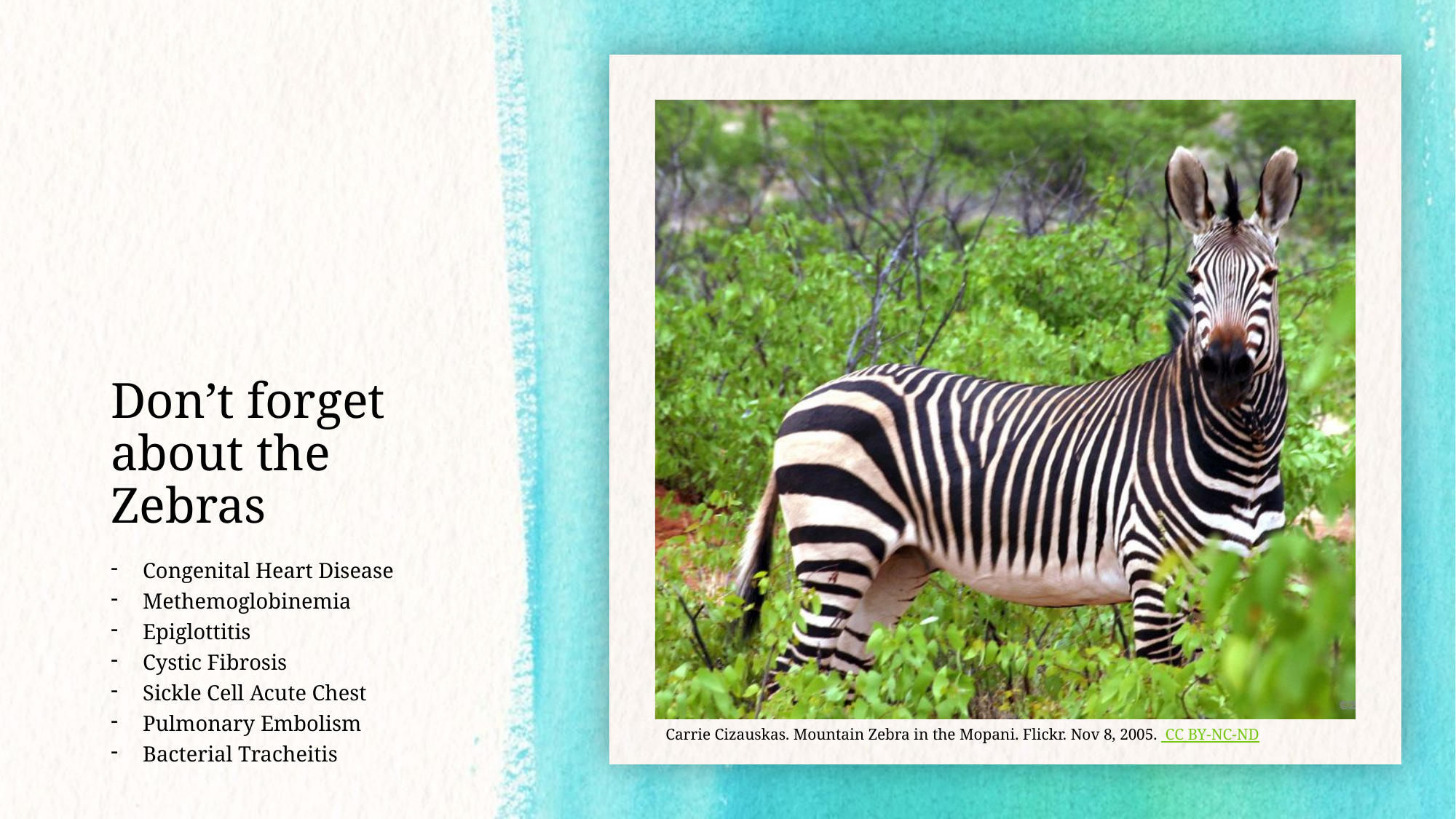

# Don’t forget about the Zebras
Congenital Heart Disease
Methemoglobinemia
Epiglottitis
Cystic Fibrosis
Sickle Cell Acute Chest
Pulmonary Embolism
Bacterial Tracheitis
Carrie Cizauskas. Mountain Zebra in the Mopani. Flickr. Nov 8, 2005. CC BY-NC-ND

## Slide 44
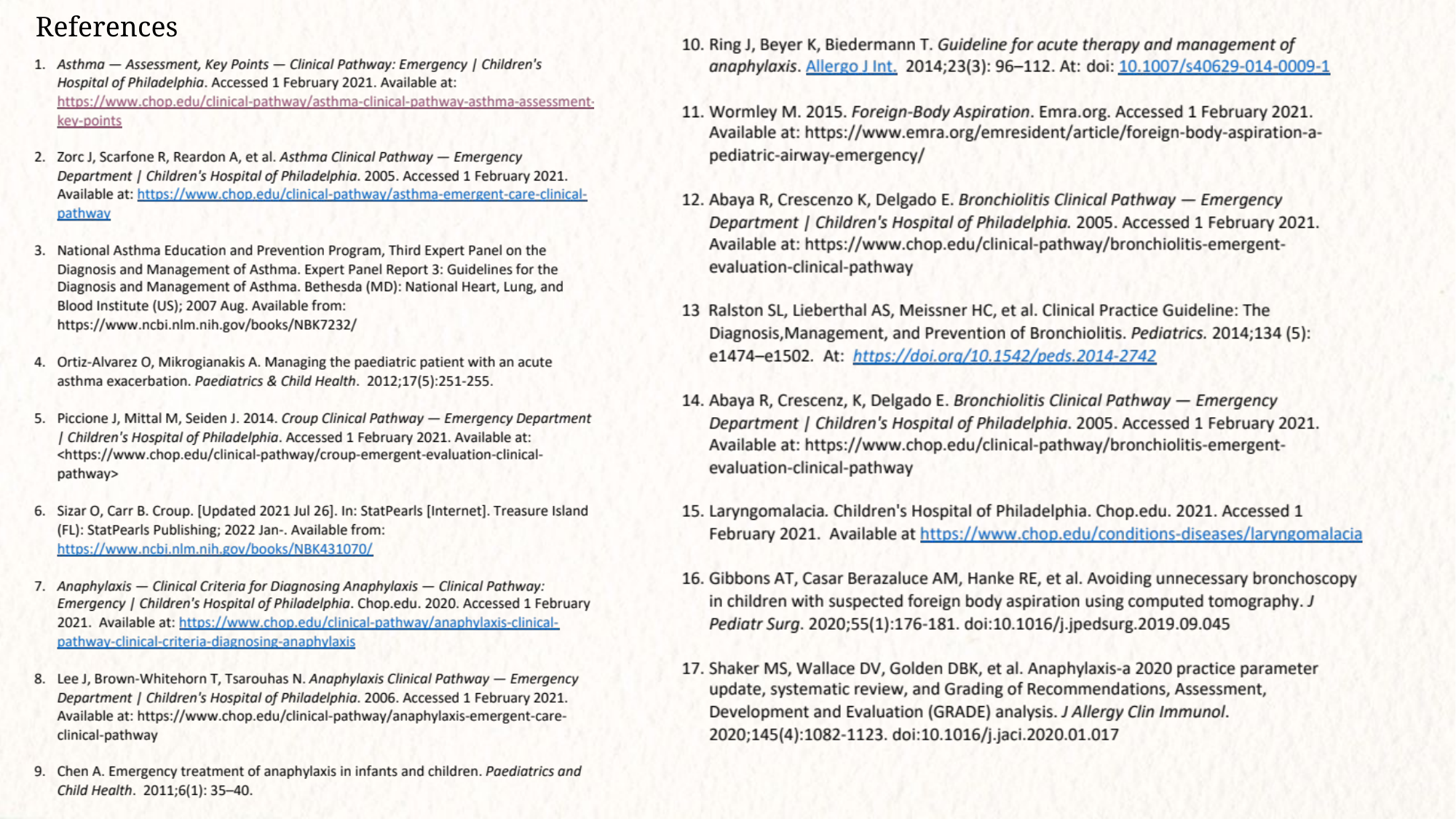

References
